# Asymmetric allylic alkylation of Morita–Baylis–Hillman carbonates with α-fluoro-β-keto esters

**DOI:** 10.3762/bjoc.9.216

**Published:** 2013-09-11

**Authors:** Lin Yan, Zhiqiang Han, Bo Zhu, Caiyun Yang, Choon-Hong Tan, Zhiyong Jiang

**Affiliations:** 1 Institute of Chemical Biology, Henan University, Jinming Campus, Kaifeng, Henan, 475004, P. R. China; 2 Key Laboratory of Natural Medicine and Immuno-Engineering of Henan Province, Henan University, P. R. China; 3 Division of Chemistry and Biological Chemistry, Nanyang Technological University, 21 Nanyang Link, Singapore 637371

**Keywords:** allylic alkylation, asymmetric catalysis, fluorine, fluoro-β-keto esters, Morita–Baylis–Hillman carbonates, natural product

## Abstract

In the presence of a commercially available *Cinchona* alkaloid as catalyst, the asymmetric allylic alkylation of Morita–Baylis–Hillman carbonates, with α-fluoro-β-keto esters as nucleophiles, have been successfully developed. A series of important fluorinated adducts, with chiral quaternary carbon centres containing a fluorine atom, was achieved in good yields (up to 93%), with good to excellent enantioselectivities (up to 96% ee) and moderate diastereoselectivities (up to 4:1 dr).

## Introduction

Fluorine is the most electronegative element in the periodic table, resulting in a highly polar C–F bond. This gives fluoro-organic compounds unique properties, compared with their parent compounds [1]. Due to the rareness of organofluorine compounds in nature, synthetic fluorinated compounds have been widely applied in numerous areas, including materials, agrochemicals, pharmaceuticals and fine chemicals [2–4]. In this context, the stereoselective introduction of fluorine atoms in molecules has become one of the most exciting and intense research areas in the recent years.

Lewis base-catalyzed asymmetric allylic alkylations (AAA) of Morita–Baylis–Hillman (MBH) adducts [5–6], such as acetates and carbonates, have become an attractive option to access various chiral *C*- [7–19], *N*- [20–25], *O*- [26–30], *P*- [31–33] and *S*-allylic [34] and spirocyclic compounds [35–37]. Several protocols have been established to introduce fluorine atoms in AAA of MBH adducts. For example, to introduce a CF_3_ group, Shibata and co-workers [13] and Jiang and co-workers [14] successively reported the asymmetric allylic trifluoromethylation of MBH adducts with Ruppert’s reagent [(trifluoromethyl)trimethylsilane, Me_3_SiCF_3_] in the presence of (DHQD)_2_PHAL as catalyst. In 2011, our research group [15], Shibata and co-workers [16] and Rios and co-workers [17] reported the addition of fluoromethyl(bisphenylsulfones) to MBH carbonates to access chiral monofluoromethyl derivatives. Furthermore, Rios and co-workers presented an asymmetric substitution of MBH carbonates with 2-fluoromalonates in good enantioselectivities [38]. Notably, the reaction between an achiral fluorocarbon nucleophile with MBH carbonates, to afford compounds with chiral quaternary carbon centres bearing a fluorine atom, remains a formidable task. Since 2009, we developed a highly enantioselective and diastereoselective guanidine-catalyzed conjugate addition and Mannich reaction of α-fluoro-β-ketoesters with excellent results [38–40]. Herein, we wish to report the first allylic alkylation of MBH carbonates with α-fluoro-β-ketoesters in excellent enantioselectivities and moderate diastereoselectivities, furnishing enantiopure fluorinated compounds with chiral quaternary carbon centres containing a fluorine atom.

## Results and Discussion

In the preliminary experiments, we investigated the reaction of α-fluoro-β-ketoester **1a** with MBH carbonate **2a** as the model substrate, in the presence of several commercially available *Cinchona* alkaloids as Lewis base catalysts (Table 1). First, the reaction was conducted in the presence of quinine at 50 °C in dichloroethane (DCE) as the solvent (Table 1, entry 1). The desired adduct **3aa** was obtained in 53% yield with poor enantio- and diastereoselectivity. Cinchonine provided similarly poor results (Table 1, entry 2). Next, we screened a series of *C*_2_-symmetric bis-*Cinchona* alkaloids as catalysts under the same conditions (Table 1, entries 3–7). (DHQD)_2_PHAL showed moderate catalytic activity; **3aa** was obtained in 67% yield with 71% ee and 60:40 dr (entry 3). The effects of solvent were then investigated (Table 1, entries 8–18). The best-performing solvent was mesitylene with respect to enantio- and diastereoselectivity; providing **3aa** in 78% yield with 89% ee and 71:29 dr (entry 18). The reaction temperature can be decreased to 25 °C and 67% yield of **3aa** with 92% ee and 74:26 dr was obtained (entry 19). A slight increase in enantio- and diastereoselectivity could be obtained when the reaction temperature was decreased to 10 °C, but the reaction rate became too sluggish to be useful (Table 1, entry 20).

**Table 1:** Catalyst screening^a^.

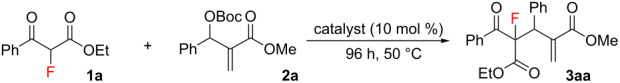					

Entry	Catalyst	Solvent	Yield (%)^b^	ee (%)^c^	dr^c^

1	quinine	DCE	53	32 (27)	55:45
2	cinchonine	DCE	59	21 (10)	55:45
3	(DHQD)_2_PHAL	DCE	67	71 (57)	60:40
4	(DHQD)_2_AQN	DCE	53	–5 (–5)	56:44
5	(DHQ)_2_PHAL	DCE	64	–35 (–1)	52:48
6	(DHQ)_2_PYR	DCE	60	–25 (–1)	59:41
7	(DHQ)_2_AQN	DCE	47	–11 (–10)	55:45
8	(DHQD)_2_PHAL	DCM	56	69 (55)	58:42
9	(DHQD)_2_PHAL	toluene	78	85 (65)	67:33
10	(DHQD)_2_PHAL	Et_2_O	59	45 (30)	55:45
11	(DHQD)_2_PHAL	EA	58	55 (30)	55:45
12	(DHQD)_2_PHAL	THF	61	31 (49)	63:37
13	(DHQD)_2_PHAL	MeCN	57	49 (19)	65:35
14	(DHQD)_2_PHAL	MeOH	63	35 (20)	60:40
15	(DHQD)_2_PHAL	*o*-xylene	74	85 (65)	72:28
16	(DHQD)_2_PHAL	*m*-xylene	65	87 (74)	70:30
17	(DHQD)_2_PHAL	*p*-xylene	72	85 (55)	68:32
18	(DHQD)_2_PHAL	mesitylene	78	89 (72)	71:29
19^d^	(DHQD)_2_PHAL	mesitylene	67	92 (69)	74:26
20^e^	(DHQD)_2_PHAL	mesitylene	45	94 (55)	75:25

^a^Unless otherwise noted, reactions were performed with 0.05 mmol of **1a**, 0.15 of **2a**, and 0.005 mmol of catalyst in 0.5 mL solvent. ^b^Yield of isolated product. ^c^Determined by HPLC methods. The data in parenthesis is the ee value of the minor diastereoisomer ^d^The reaction was conducted at 25 °C, 1.0 mmol scale in 1.0 mL of mesitylene. ^e^The reaction was conducted at 10 °C, 1.0 mmol scale in 1.0 mL of mesitylene.

Using the established conditions, allylic alkylations of α-fluoro-β-ketoesters **1b–g** with MBH carbonate **2a** were found to afford the products **3ba–ga** in 67–79% yield with 88–96% ee and 3:1 to 4:1 dr (Table 2, entries 1–6). The results showed that the introduction of various aryl substituents in α-fluoro-β-ketoesters did not affect the reactivity and stereoselectivity. Subsequently, the scope of the allylic alkylation with respect to various MBH carbonates **2** and α-fluoro-β-ketoester **1a** was investigated (Table 2, entries 7–20). The desired allylic alkylation adducts **3ab–o** were achieved in moderate to good yields with good to excellent enantioselectivities and moderate diastereoselectivities. MBH carbonates (Table 2, **2b–k**) with electron-withdrawing groups appended on the aromatic rings were more active than those (Table 2, **2l–m**) with electron-neutral and donating groups. Excellent ee values with moderate dr values were obtained when the phenyl groups of MBH carbonates were replaced with hetereoaromatic groups, such as thiophene and furan (Table 2, **2n–o**).

**Table 2:** Allylic alkylation of α-fluoro-β-ketoesters **1** with MBH carbonates **2**^a^.

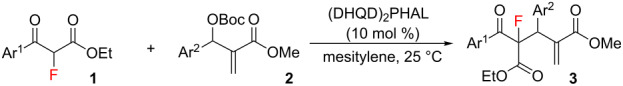							

Entry	Ar^1^, **1**	Ar^2^, **2**	Time (h)	**3**	Yield (%)^b^	ee (%)^c^	dr^d^

1	*p*-FPh, **1b**	Ph, **2a**	40	**3ba**	71	88	3:1
2	*p*-ClPh, **1c**	Ph, **2a**	70	**3ca**	79	93	3:1
3	*p*-BrPh, **1d**	Ph, **2a**	70	**3da**	75	96	3:1
4	*m*-BrPh, **1e**	Ph, **2a**	70	**3ea**	72	90	3:1
5	3,5-Cl_2_Ph, **1f**	Ph, **2a**	70	**3fa**	69	88	3:1
6	*p*-MePh, **1g**	Ph, **2a**	50	**3ga**	67	94	4:1
7	Ph, **1a**	*p*-NO_2_Ph, **2b**	70	**3ab**	91	95	3:1
8	Ph, **1a**	*p*-CF_3_Ph, **2c**	70	**3ac**	65	87	4:1
9	Ph, **1a**	*p*-FPh, **2d**	70	**3ad**	71	90	3:1
10	Ph, **1a**	*p*-ClPh, **2e**	70	**3ae**	73	93	4:1
11	Ph, **1a**	*p*-BrPh, **2f**	96	**3af**	64	91	4:1
12	Ph, **1a**	*m*-NO_2_Ph, **2g**	96	**3ag**	93	95	3:1
13	Ph, **1a**	*m*-ClPh, **2h**	70	**3ah**	81	91	3:1
14	Ph, **1a**	*m*-BrPh, **2i**	70	**3ai**	78	90	4:1
15	Ph, **1a**	*o*-FPh, **2j**	96	**3aj**	73	86	4:1
16	Ph, **1a**	*o*-ClPh, **2k**	70	**3ak**	84	86	4:1
17	Ph, **1a**	*p*-MePh, **2l**	70	**3al**	53	91	4:1
18	Ph, **1a**	*p*-MeOPh, **2m**	70	**3am**	50	91	3:1
19	Ph, **1a**	2-thienyl, **2n**	90	**3an**	78	92	4:1
20	Ph, **1a**	2-furyl, **2o**	96	**3ao**	73	84	3:1

^a^Reactions were performed with 0.1 mmol of **1**, 0.3 mmol of **2**, and 0.005 mmol of (DHQD)_2_PHAL in 1.0 mL mesitylene. ^b^Yield of isolated product. ^c^Determined by chiral HPLC on the major diastereoisomer. ^d^Determined by ^1^H NMR analysis.

## Conclusion

We have developed an asymmetric allylic alkylation of MBH carbonates with α-fluoro-β-ketoesters, catalyzed by a commercially available *Cinchona* alkaloid. Several fluorinated adducts, with chiral quaternary carbon centres containing a fluorine atom, were successfully prepared in 50–93% yields with 84–96% ee and a dr of 3:1 to 4:1. The absolute configurations of adducts still have to be determined and will be reported in due course.

## Experimental

**Representative procedure for the synthesis of 3aa**: α-Fluoro-β-ketoester **1a** (21.0 mg, 1.0 equiv, 0.1 mmol) and (DHQD)_2_PHAL (7.8 mg, 0.1 equiv, 0.01 mmol) were dissolved in mesitylene (1.0 mL) at 25 °C. After the addition of MBH carbonate **2a** (3.0 equiv, 0.3 mmol) the reaction mixture was stirred at 25 °C. The reaction was monitored by TLC. After 96 hours, flash chromatography affords product **3aa** (25.7 mg, 67% yield) as colorless oil.

## Supporting Information

File 1 Experimental details and spectroscopic data.
