# Supplementary material for: Asymmetric allylic alkylation of Morita–Baylis–Hillman carbonates with α-fluoro-β-keto esters
Source: Beilstein J Org Chem. 2013 Sep 11;9:1853–7. doi: 10.3762/bjoc.9.216 (PMC3778419; doi:10.3762/bjoc.9.216)
Supplement: File 1 — Experimental details and spectroscopic data. [file Beilstein_J_Org_Chem-09-1853-s001.pdf]

Supporting Information

for

Asymmetric allylic alkylation of Morita–Baylis–Hillman carbonates with α-fluoro-β-keto esters

Lin Yan,[1] Zhiqiang Han,[1] Bo Zhu,[1] Caiyun Yang,[1] Choon-Hong Tan[2,3,*] and Zhiyong Jiang[1,2,3,*]

Address: [1] Institute of Chemical Biology, Henan University, Jinming Campus, Kaifeng, Henan, 475004, P. R. China; [2] Key Laboratory of Natural Medicine and Immuno-Engineering of Henan Province, Henan University, P. R. China; [3] Division of Chemistry and Biological Chemistry, Nanyang Technological University, 21 Nanyang Link, Singapore 637371

Email: Zhiyong Jiang - chmjzy@henu.edu.cn; Choon-Hong Tan - choonhong@ntu.edu.sg

* Corresponding author

Experimental details and spectroscopic data.

Table of Contents

1 General Information s2

2 Representative procedure for the synthesis of **3aa** s4

3 Characterization of Adducts s5

4 Copies of NMR Spectra s21

1 General information

General procedures and methods

Experiments involving moisture and/or air sensitive components were performed under a positive pressure of nitrogen in oven-dried glassware equipped with a rubber septum inlet. Dried solvents and liquid reagents were transferred by oven-dried syringes or hypodermic syringes cooled to ambient temperature in a desiccator. Reaction mixtures were stirred in 4 mL sample vials with Teflon-coated magnetic stirring bars unless otherwise stated. Moisture in non-volatile reagents/compounds was removed in high vacuo by means of an oil pump and subsequent purging with nitrogen. Solvents were removed in vacuo under ~30 mmHg and heated with a water bath at 30–50 °C using a rotary evaporator with aspirator. The condenser was cooled with running water at 0 °C.

All experiments were monitored by analytical thin layer chromatography (TLC). TLC was performed on precoated plates, 60 F_{254}. After elution, plates were visualized under UV illumination at 254 nm for UV active material. Further visualization was achieved by staining with $KMnO_4$, ceric molybdate, or anisaldehyde solution. For those using the aqueous stains, the TLC plates were heated on a hot plate.

Columns for flash chromatography (FC) contained silica gel 200–300 mesh. Columns were packed as slurry of silica gel in petroleum ether and equilibrated solution using the appropriate solvent system. The elution was assisted by applying pressure of about 2 atm with an air pump.

Instrumentations

Proton nuclear magnetic resonance (^1H NMR) and carbon NMR (^{13}C NMR), spectra were recorded in $CDCl_3$ unless otherwise stated. ^1H (400 MHz), and ^{13}C (100 MHz) were performed on a 400 MHz NMR spectrometer. Chemical shifts are reported in parts per million (ppm), using the residual solvent signal as an internal standard: $CDCl_3$ (^1H NMR: δ 7.26, singlet; ^{13}C NMR: δ 77.0, triplet). HRMS (Analyzer: TOF) was reported in units of mass of charge ratio (m/z). Mass samples were dissolved in CH_3CN (HPLC Grade) unless otherwise stated. Optical rotations were recorded on a polarimeter with a sodium lamp at a wavelength of 589 nm and reported as follows:

$[\alpha]_{\lambda}^{T\,°C}$ (*c* = g/100 mL, solvent).

Enantiomeric excesses were determined by chiral High Performance Liquid Chromatography (HPLC) analysis. HPLC samples were dissolved in HPLC grade isopropanol (IPA) unless otherwise stated.

Materials

All commercial reagents were purchased with the highest purity grade. They were used without further purification unless specified. All solvents used, mainly petroleum ether (PE) and ethyl acetate (EtOAc), were distilled. Anhydrous DCM and MeCN were freshly distilled from CaH_2 and stored under N_2 atmosphere. THF, Et_2O, toluene, *p*-xylene and *m*-xylene were freshly distilled from sodium/benzophenone before used.

2 Representative procedure for the synthesis of 3aa

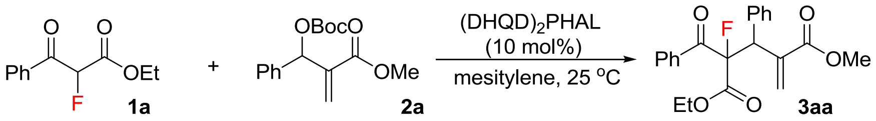

α-Fluoro-β-ketoester **1a** (21.0 mg, 1.0 equiv, 0.1 mmol) and $(DHQD)_2PHAL$ (7.8 mg, 0.1 equiv, 0.01 mmol) were dissolved in mesitylene (1.0 mL) at 25 °C. After MBH carbonate **2a** (3.0 equiv, 0.3 mmol) was added, the reaction mixture was stirred at 25 °C and monitored by TLC. After 96 hours, flash chromatography affords product **3aa** (25.7 mg, 67% yield) as a colorless oil.

3. Characterization of adducts

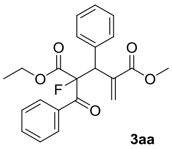

3aa

Colorless oil, 67% yield; 92% ee; 3:1 dr; ^{1}H NMR (400 MHz, $CDCl_3$) δ 8.07–7.68 (m, 2H), 7.48–7.28 (m, 5H), 7.24–7.13 (m, 2H), 6.52–5.96 (m, 2H), 5.55–5.32 (m, 1H), 4.38–4.03 (m, 2H), 3.71–3.70 (m, 3H), 1.27–1.07 (m, 3H). ^{13}C NMR (100 MHz, $CDCl_3$) δ 193.0, 192.7, 166.4, 166.1, 165.8, 138.5, 135.3, 135.2, 134.8, 133.9, 133.3, 130.6, 130.6, 120.0, 129.9, 129.8, 129.5, 129.4, 128.6, 128.4, 128.2, 127.9, 127.5, 127.3, 127.2, 126.1, 104.4, 102.4, 63.0, 63.0, 52.2, 49.2, 49.0, 13.9, 13.8. HRMS (ESI) *m/z* 384.1365 (M^+), calcd. for $C_{22}H_{21}FO_5$ 384.1373.

The ee was determined by HPLC analysis. Lux 5u cellulose-3 (250 x 4.6 mm); hexane/2-propanol = 80/20; flow rate 1.0 mL/min; 25 °C; 254 nm; retention time: 6.3 min (major) and 9.8 min (minor).

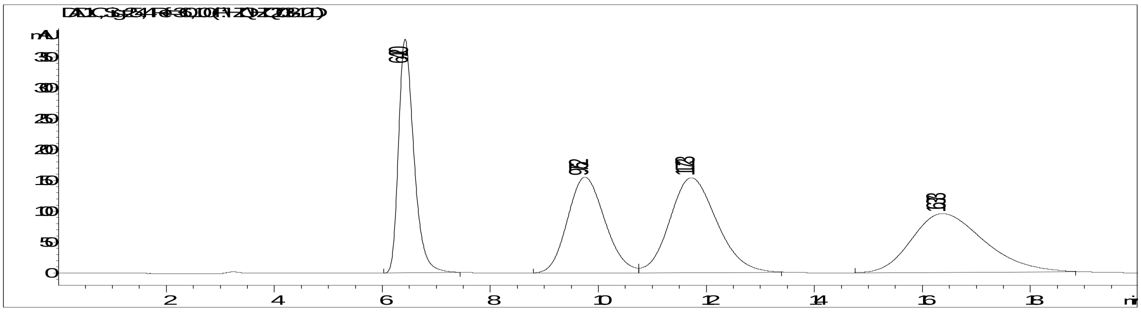

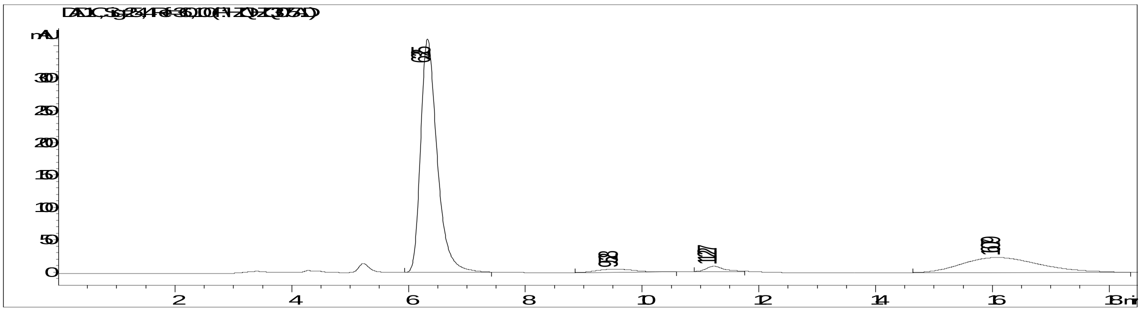

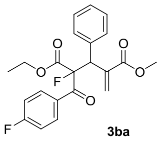

3ba

Colorless oil, 71% yield; 88% ee; 3:1 dr; ^{1}H NMR (400 MHz, $CDCl_3$) δ 8.10–7.76 (m, 2H), 7.42–7.27 (m, 3H), 7.24–7.09 (m, 3H), 7.01–6.95 (m, 1H), 6.55–5.94 (m, 2H), 5.53–5.31 (m, 1H), 4.36–4.02 (m, 2H), 3.70–3.70 (m, 3H), 1.25–1.07 (m, 3H). ^{13}C NMR (100 MHz, $CDCl_3$) δ 192.7, 192.4, 166.3, 165.7, 160.9, 138.5, 134.6, 134.0, 133.5, 132.4, 132.3, 131.7, 130.9, 129.8, 129.6, 129.5, 128.6, 128.3, 127.2, 127.1, 115.4, 115.1, 114.9, 100.0, 63.1, 52.3, 48.4, 48.2, 13.9. HRMS (ESI) *m/z* 425.1178 ($M + Na^+$), calcd. for $C_{22}H_{20}F_2O_5Na$ 425.1171.

The ee was determined by HPLC analysis. Lux 5u cellulose-4 (250 x 4.6 mm); Hexane/2-propanol = 90/10; flow rate 1.0 mL/min; 25 °C; 254 nm; retention time: 6.8 min (major) and 9.3 min (minor).

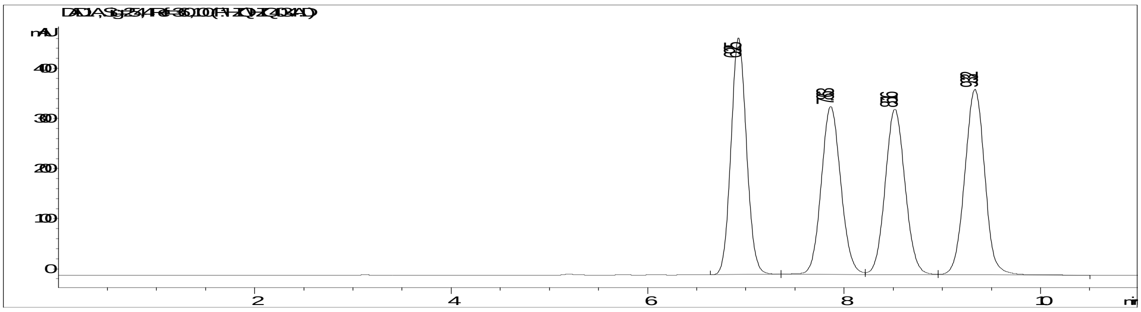

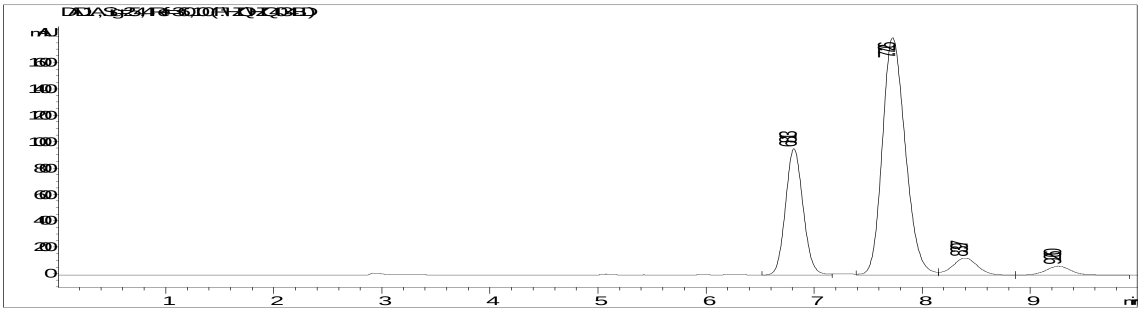

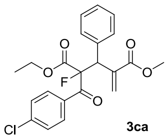

3ca

Colorless oil, 79% yield; 93% ee; 3:1 dr; ^{1}H NMR (400 MHz, $CDCl_3$) δ 8.08–7.87 (m, 1H), 7.66–7.58 (m, 1H), 7.45–7.26 (m, 5H), 7.24–7.14 (m, 2H), 6.53–5.95 (m, 2H), 5.52–5.30 (m, 1H), 4.42–4.01 (m, 2H), 3.70 (s, 3H), 1.24–1.10 (m, 3H). ^{13}C NMR (100 MHz, $CDCl_3$) δ 192.0, 191.8, 166.3, 165.9, 165.6, 140.0, 138.4, 135.1, 135.0, 132.9, 132.9, 131.4, 131.3, 131.0, 130.9, 130.5 (two peaks), 130.0, 129.9, 129.0, 128.5, 128.4, 128.2, 128.0, 127.6, 127.2, 127.2, 104.5, 102.4, 63.2, 52.3, 49.2, 49.0, 29.7, 27.7, 13.8. HRMS (ESI) *m/z* 441.0883 (M + Na^+), calcd. for $C_{22}H_{20}ClFO_5Na$ 441.0876. The ee was determined by HPLC analysis. Lux 5u cellulose-3 (250 x 4.6 mm); hexane/2-propanol = 90/10; flow rate 1.0 mL/min; 25 ^{o}C; 254 nm; retention time: 4.7 min (major) and 5.2 min (minor).

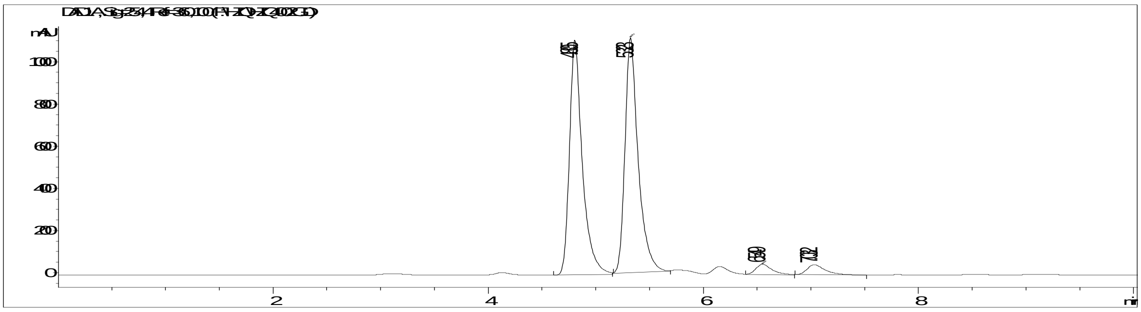

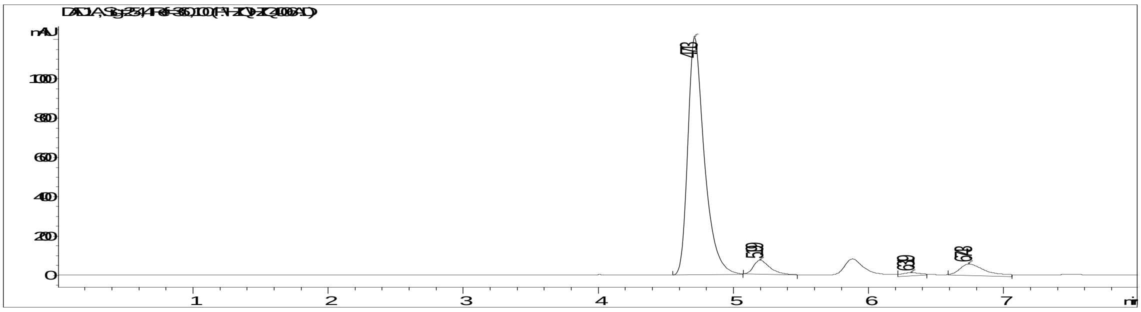

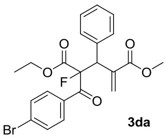
3da

Colorless oil, 75% yield; 96% ee; 3:1 dr; ^{1}H NMR (400 MHz, $CDCl_3$) δ 7.94–7.27 (m, 7H), 7.24–7.13 (m, 2H), 6.51–5.94 (m, 2H), 5.51–5.30 (m, 1H), 4.39–4.02 (m, 2H), 3.71–3.70 (m, 3H), 1.26–1.07 (m, 3H). ^{13}C NMR (100 MHz, $CDCl_3$) δ 192.3, 192.0, 190.7, 166.5, 166.3, 165.8, 165.6, 141.5, 139.1, 138.4, 135.1, 135.0, 133.4, 132.9, 132.0, 131.6, 131.4, 131.3, 131.0, 131.0, 130.5, 129.9, 129.6, 128.8, 128.4, 128.30, 128.2, 128.0, 127.6, 127.2, 127.2, 126.1, 124.8, 104.5, 102.4, 63.2, 60.4, 52.3, 51.6, 49.2, 49.0, 21.0, 14.2, 13.9, 13.8. HRMS (ESI) *m/z* 485.0372 (M + Na^+), calcd. for $C_{22}H_{20}BrFO_5Na$ 485.0370.

The ee was determined by HPLC analysis. Lux 5u cellulose-3 (250 x 4.6 mm); hexane/2-propanol = 80/20; flow rate 1.0 mL/min; 25 °C; 254 nm; retention time: 5.5 min (major) and 6.3 min (minor).

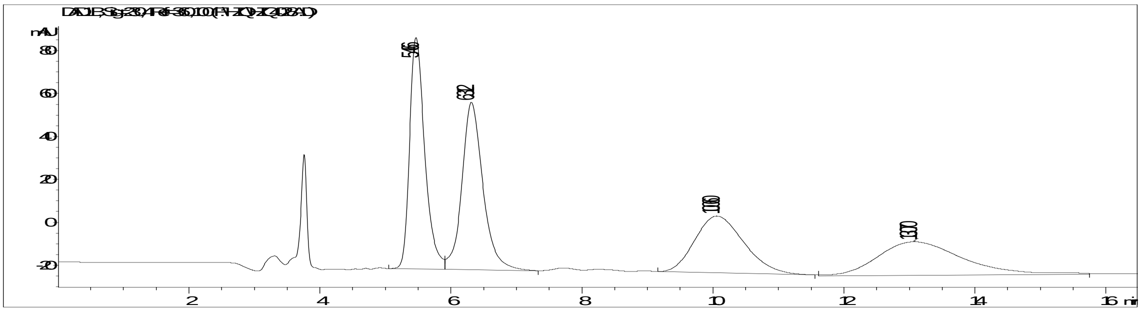

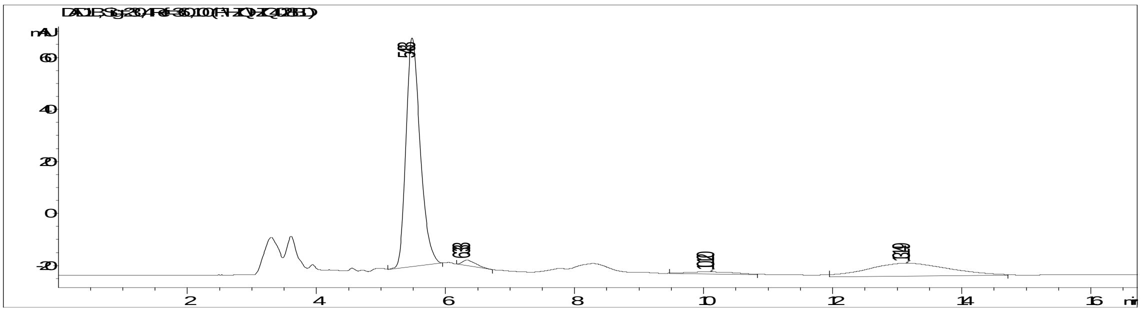

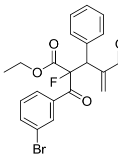
3ea

Colorless oil, 72% yield; 90% ee; 3:1 dr; ^{1}H NMR (400 MHz, $CDCl_3$) δ 8.15–7.29 (m, 7H), 7.24–7.16 (m, 2H), 6.49–5.96 (m, 2H), 5.50–5.30 (m, 1H),

4.41–4.01 (m, 2H), 3.71–3.70 (m, 3H), 1.27–1.08 (m, 3H).^{13}C NMR (100 MHz, $CDCl_3$) δ 192.1, 191.8, 166.4, 166.3, 165.6, 165.4, 139.0, 138.4, 136.8, 136.3, 136.1, 135.0, 134.8, 132.6, 132.3, 132.2, 130.5, 130.2, 129.9, 129.7, 128.4, 128.3, 128.0, 127.7, 127.2, 127.1, 126.2, 122.3, 104.4, 102.4, 63.2, 52.2, 49.3, 49.1, 13.8. HRMS (ESI) *m/z* 485.0373 (M + Na^+), calcd. for $C_{22}H_{20}BrFO_5Na$ 485.0370.

The ee was determined by HPLC analysis. CHIRALPAK IB (4.6 mm i.d. x 250 mm); hexane/2-propanol = 97/03; flow rate 0.3 mL/min; 25 °C; 254 nm; retention time: 33.8 min (major).

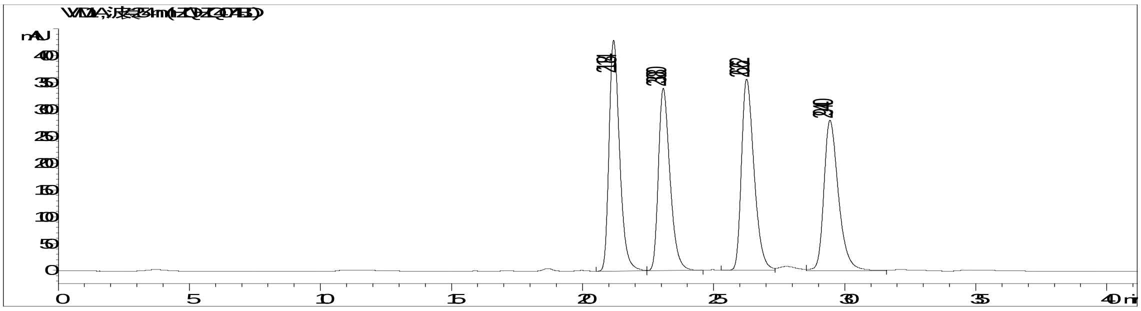

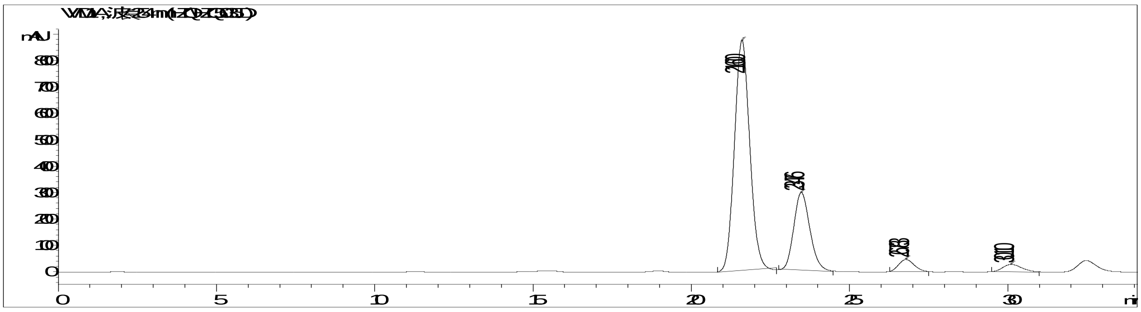

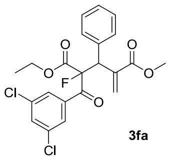

3fa

Colorless oil, 69% yield; 88% ee; 3:1 dr; ^{1}H NMR (400 MHz, $CDCl_3$) δ 7.91–7.27 (m, 6H), 7.25–7.15 (m, 2H), 6.54–5.93 (m, 2H), 5.48–5.16 (m, 1H), 4.39–3.99 (m, 2H), 3.71–3.70 (m, 3H), 1.27–1.08 (m, 3H). ^{13}C NMR (100 MHz, $CDCl_3$) δ 191.3, 191.0, 166.2, 165.5, 165.3, 138.3, 138.1, 134.7, 134.0, 132.9, 131.7, 131.4, 131.4, 130.8, 130.5, 130.3, 129.9, 128.9, 128.6, 128.5, 128.4, 128.3, 128.0, 128.0, 127.8, 127.1, 127.1, 104.5, 102.5, 63.3, 52.3, 49.3, 49.1, 13.8, 13.8. HRMS (ESI) *m/z* 475.0492 (M + Na^+), calcd. for $C_{22}H_{19}Cl_2FO_5Na$ 475.0486.

The ee was determined by HPLC analysis. CHIRALPAK IB (4.6 mm i.d. x 250 mm); hexane/2-propanol = 97/03; flow rate 0.3 mL/min; 25 ^{o}C; 254 nm; retention time: 21.6 min (major) and 26.8 min (minor).

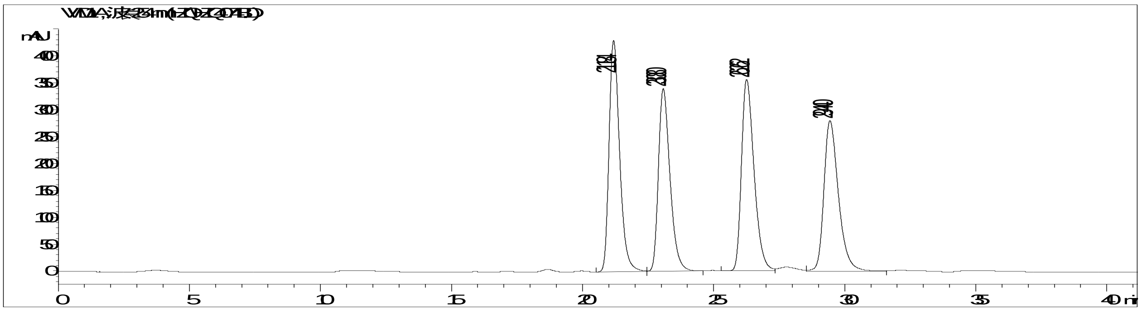

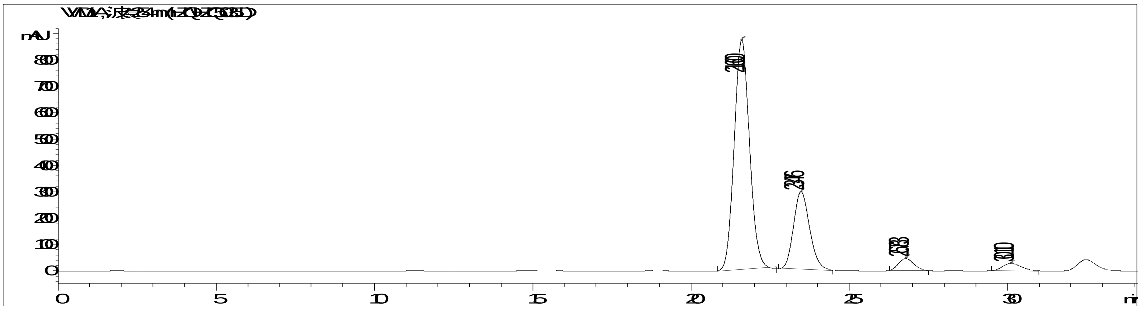

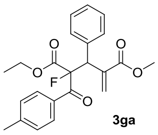

3ga

Colorless oil, 67% yield; 94% ee; 3:1 dr; ^{1}H NMR (400 MHz, $CDCl_3$) δ 7.96–7.65 (m, 2H), 7.37–7.35 (m, 2H), 7.29 (d, *J* = 7.5 Hz, 1H), 7.24–7.10 (m, 4H), 6.55–5.90 (m, 2H), 5.55–5.30 (m, 1H), 4.37–4.02 (m, 2H), 3.70 (s, 3H), 2.40–2.32 (m, 3H), 1.25–1.06 (m, 3H). ^{13}C NMR (100 MHz, $CDCl_3$) δ 192.2, 192.0, 166.6, 166.4, 166.2, 166.0, 145.1, 144.4, 139.2, 138.6, 135.4, 135.3, 132.1, 132.0, 130.6, 130.1, 130.0, 130.0, 129.70, 129.3, 128.9, 128.3, 128.1, 127.8, 127.4, 127.3, 127.2, 126.0, 104.4, 102.4, 62.9, 62.9, 60.4, 52.2, 49.0, 48.8, 21.7, 21.6, 21.0, 14.2, 13.8, 13.8. HRMS (ESI) m/z 421.1431 (M + Na^+), calcd. for $C_{23}H_{23}FO_5$ Na 421.1422.

The ee was determined by HPLC analysis. Lux 5u cellulose-3 (250 x 4.6 mm); hexane/2-propanol = 90/10; flow rate 1.0 mL/min; 25 °C; 254 nm; retention time: 9.6 min (major) and 11.5 min (minor).

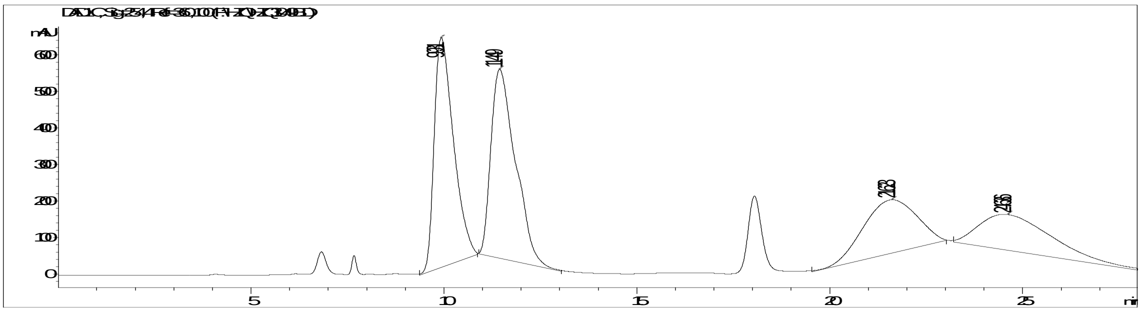

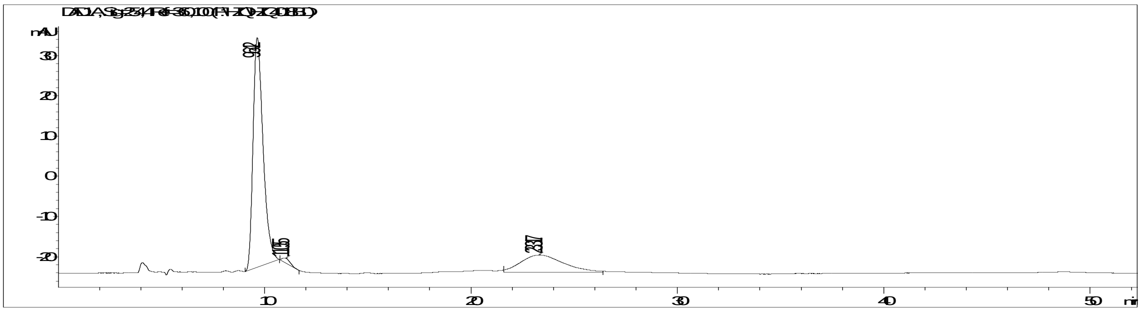

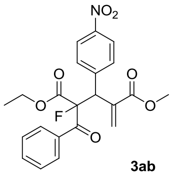

3ab

Colorless oil, 91% yield; 95% ee; 3:1 dr; ^{1}H NMR (400 MHz, $CDCl_3$) δ 8.18–7.83 (m, 4H), 7.66–7.31 (m, 5H), 6.56–6.02 (m, 2H), 5.68–5.63 (m, 1H), 4.40–4.05 (m, 2H), 3.72 (s, 3H), 1.25–1.09 (m, 3H). ^{13}C NMR (100 MHz, $CDCl_3$) δ 191.6, 191.3, 190.9, 190.6, 166.1, 166.0, 165.6, 165.3, 147.6, 147.2, 143.1, 143.0, 138.1, 137.5, 134.3, 134.0, 133.9, 133.9, 131.6, 131.6, 131.0 (two peaks), 129.9, 129.8, 129.8, 129.7, 128.7, 128.5, 128.4, 127.3, 127.2, 123.8, 123.5, 123.2, 103.8, 101.8, 63.4, 53.4, 52.4, 52.4, 49.0, 48.9, 48.5, 48.3, 13.8. HRMS (ESI) *m/z* 430.1285 ($M+H^+$), calcd. for $C_{22}H_{21}NFO_7$ 430.1297.

The ee was determined by HPLC analysis. Lux 5u cellulose-3 (250 x 4.6 mm); Hexane/2-propanol = 80/20; flow rate 1.0 mL/min; 25 °C; 254 nm; retention time: 10.5 min (major) and 17.2 min (minor).

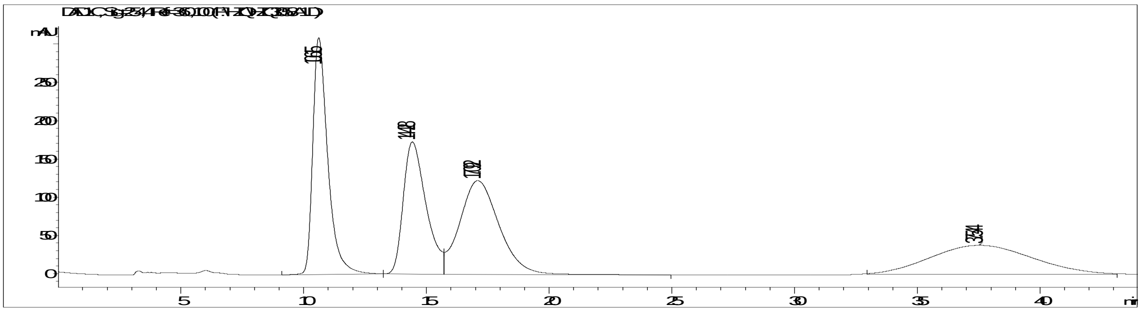

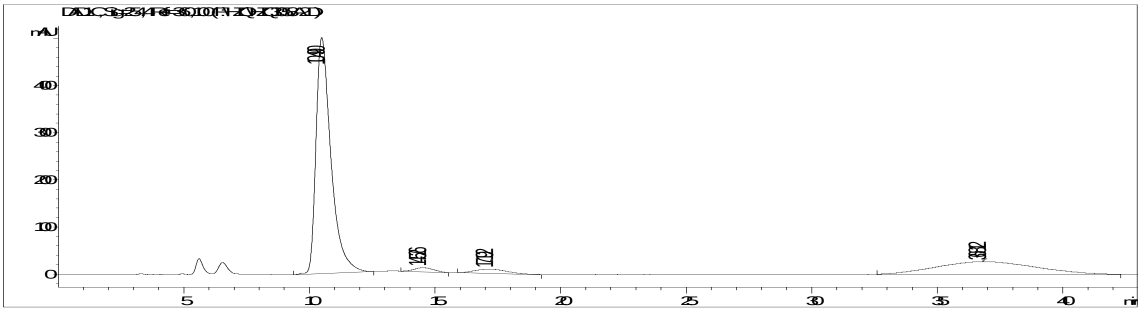

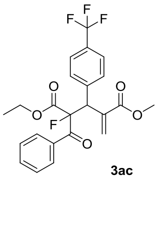

3ac

Colorless oil, 65% yield; 87% ee; 3:1 dr; ^{1}H NMR (400 MHz, $CDCl_3$) δ 8.07–7.76 (m, 2H), 7.64–7.43 (m, 6H), 7.35 (t, *J* = 7.8 Hz, 1H), 6.56–5.99 (m,

2H), 5.63–5.39 (m, 1H), 4.39–4.04 (m, 2H), 3.71 (s, 3H), 1.25–1.07 (m, 3H). ^{13}C NMR (100 MHz, $CDCl_3$) δ 192.0, 191.7, 191.2, 166.2, 166.1, 165.8, 165.6, 139.6, 138.5, 137.9, 134.2, 134.2, 134.1, 133.7, 131.0 (two peaks), 130.4, 129.9, 129.8, 129.7, 129.6, 129.5, 128.7, 128.3, 128.0, 128.0, 127.8, 126.8, 126.7, 125.7, 125.3, 125.3, 125.3, 125.1, 125.0, 125.0, 122.6, 104.1, 102.0, 63.2, 63.2, 52.4, 49.1, 48.9, 48.6, 48.4, 13.8. HRMS (ESI) *m/z* 475.1139 (M + Na^+), calcd. for $C_{23}H_{20}F_4O_5Na$ 475.114.

The ee was determined by HPLC analysis. Lux 5u cellulose-3 (250 x 4.6 mm); hexane/2-propanol = 80/20; flow rate 1.0 ml/min; 25 ^{o}C; 254 nm; retention time: 6.4 min (major) and 7.1 min (minor).

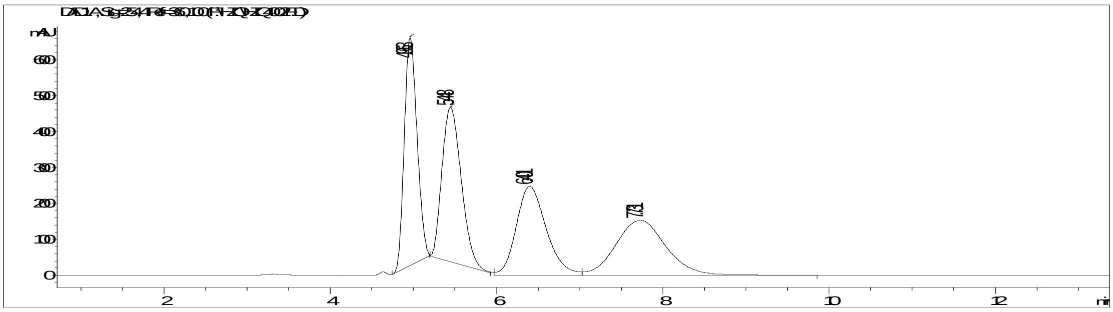

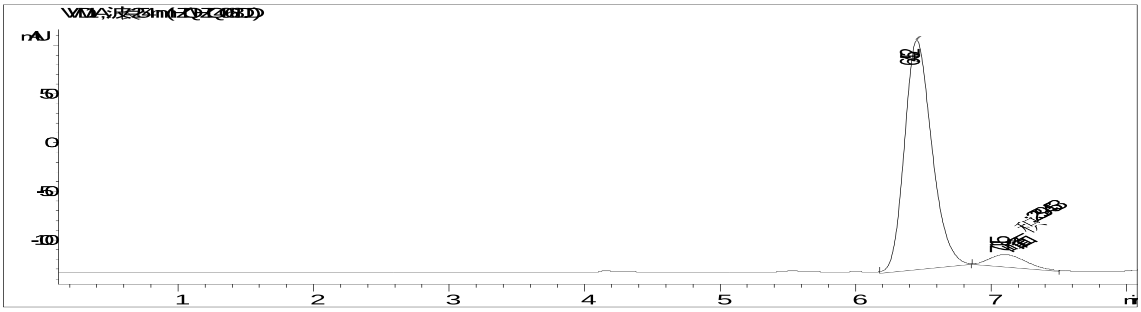

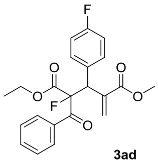

3ad

Colorless oil, 71% yield; 90% ee; 3:1 dr; ^{1}H NMR (400 MHz, $CDCl_3$) δ 8.05–7.72 (m, 2H), 7.63–7.30 (m, 5H), 7.02–63.87 (m, 2H), 6.59–5.87 (m, 2H), 5.54–5.31 (m, 1H), 4.42–4.03 (m, 2H), 3.71 (s, 3H), 1.25–1.10 (m, 3H). ^{13}C NMR (100 MHz, $CDCl_3$) δ 192.7, 192.4, 166.3, 165.7, 160.9, 138.5, 134.6, 134.0, 133.5, 132.4, 132.3, 131.7, 130.9, 129.8, 129.6, 129.5, 128.6, 128.3, 127.2, 127.1, 115.4, 115.1, 114.9, 100.0, 63.1, 52.3, 48.4, 48.2, 13.9. HRMS (ESI) *m/z* 430.1348 (M + H^+), calcd. for $C_{22}H_{21}NF_2O_5$ 403.1352.

The ee was determined by HPLC analysis. Lux 5u cellulose-3 (250 x 4.6 mm); hexane/2-propanol = 80/20; flow rate 1.0 mL/min; 25 °C; 254 nm; retention time: 4.8 min (major) and 6.5 min (minor).

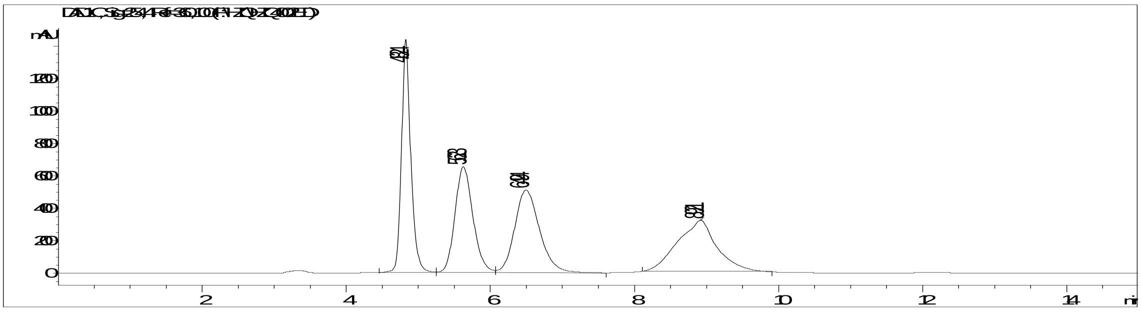

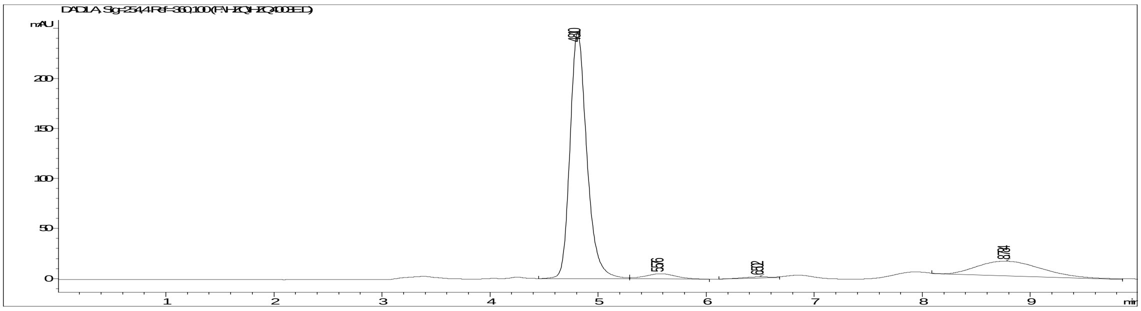

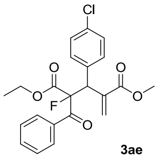

3ae

Colorless oil, 73% yield; 93% ee; 4:1 dr; ^{1}H NMR (400 MHz, $CDCl_3$) δ 8.05–7.75 (m, 2H), 7.62–7.27 (m, 6H), 7.18 (d, J = 8.5 Hz, 1H), 6.57–5.91 (m, 2H), 5.54–5.30 (m, 1H), 4.40–4.03 (m, 2H), 3.71 (s, 3H), 1.25–1.10 (m, 3H). ^{13}C NMR (100 MHz, $CDCl_3$) δ 192.4, 192.1, 166.2, 165.9, 165.6, 138.2, 134.4, 134.1, 133.8, 133.6, 133.5, 132.0 (two peaks), 131.3, 129.9, 129.8, 129.6, 129.6, 128.6, 128.6, 128.3, 127.5, 127.4, 104.2, 102.1, 63.1, 52.3, 48.4, 48.2, 13.8.

The ee was determined by HPLC analysis. Lux 5u cellulose-3 (250 x 4.6 mm); hexane/2-propanol = 80/20; flow rate 1.0 mL/min; 25 °C; 254 nm; retention time: 4.9 min (major) and 5.6 min (minor).

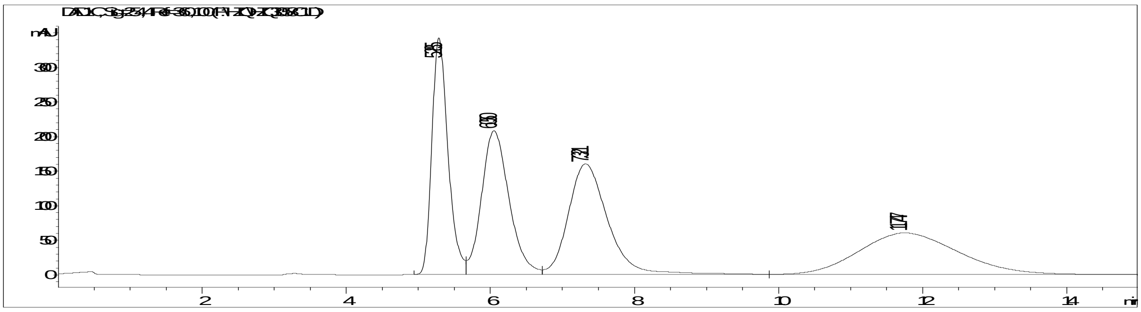

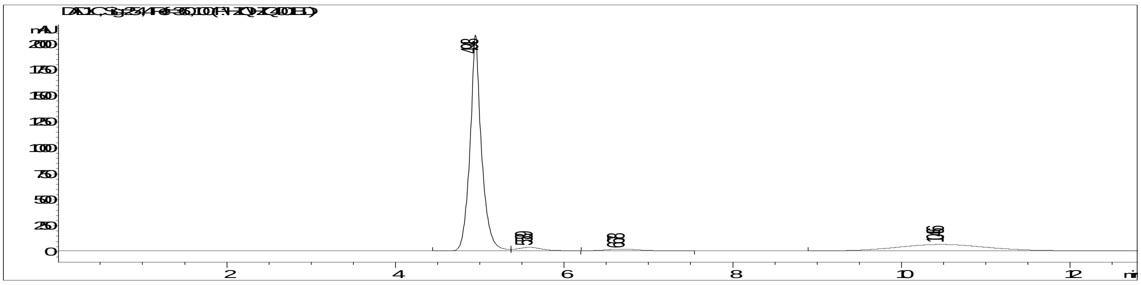

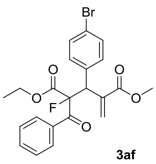

3af

Colorless oil, 64% yield; 91% ee; 4:1 dr; ^{1}H NMR (400 MHz, $CDCl_3$) δ 8.07–7.78 (m, 2H), 7.65–7.43 (m, 2H), 7.37 (t, *J* = 8.5 Hz, 3H), 7.31–7.26 (m, 2H), 6.58–5.93 (m, 2H), 5.55–5.31 (m, 1H), 4.44–4.04 (m, 2H), 3.73 (s, 3H), 1.27–1.12 (m, 3H). ^{13}C NMR (100 MHz, $CDCl_3$) δ 192.3, 192.0, 166.3, 166.2, 165.9, 165.6, 138.1, 134.4, 134.1, 133.6, 132.3, 131.6, 131.5, 131.3, 129.8, 129.8, 129.6, 129.6, 128.6, 128.3, 127.5, 127.4, 126.3, 122.2, 121.8, 104.1, 102.0, 63.12, 52.3, 48.7, 48.4, 48.2, 13.8. HRMS (ESI) *m/z* 485.0383 (M + Na^+), calcd. for $C_{22}H_{20}BrFO_5Na$ 485.0370.

The ee was determined by HPLC analysis. Lux 5u cellulose-3 (250 x 4.6 mm); hexane/2-propanol = 80/20; flow rate 1.0 mL/min; 25 °C; 254 nm; retention time: 5.2 min (major) and 5.9 min (minor).

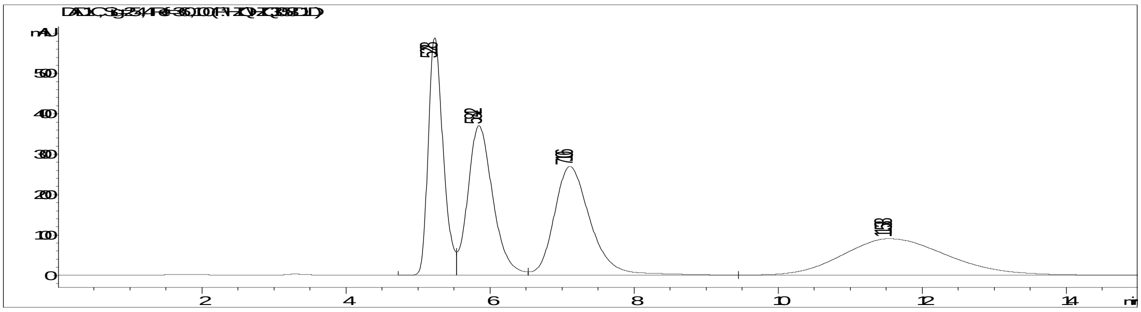

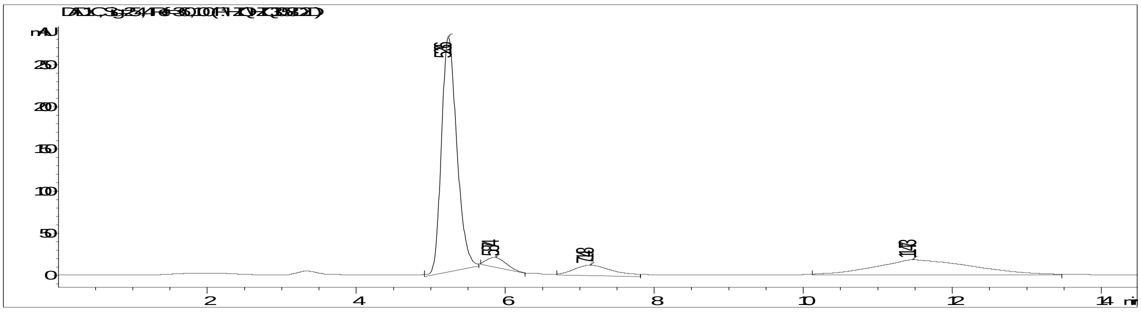

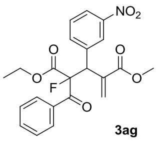

3ag

Colorless oil, 93% yield; 95% ee; 3:1 dr; ^{1}H NMR (400 MHz, $CDCl_3$) δ 8.29–7.72 (m, 5H), 7.65–7.32 (m, 4H), 6.64–6.01 (m, 2H), 5.69–5.44 (m, 1H), 4.39–4.04 (m, 2H), 3.72 (s, 3H), 1.25–1.13 (m, 3H). ^{13}C NMR (100 MHz, $CDCl_3$) δ 191.2, 166.0, 165.4, 148.1, 137.7, 137.6, 137.2, 136.2, 134.3, 134.0, 129.9, 129.8, 129.8, 129.3, 129.0, 128.7, 128.5, 128.4, 125.4, 123.1, 122.7, 101.8, 63.4, 63.4, 52.5, 48.3, 48.2, 13.9. HRMS (ESI) *m/z* 430.1289 (M + H^+), calcd. for $C_{22}H_{21}NFO_7$ 430.1297.

The ee was determined by HPLC analysis. Lux 5u cellulose-3 (250 x 4.6 mm); hexane/2-propanol = 90/10; flow rate 1.0 mL/min; 25 °C; 254 nm; retention time: 11.9 min (major) and 15.1 min (minor).

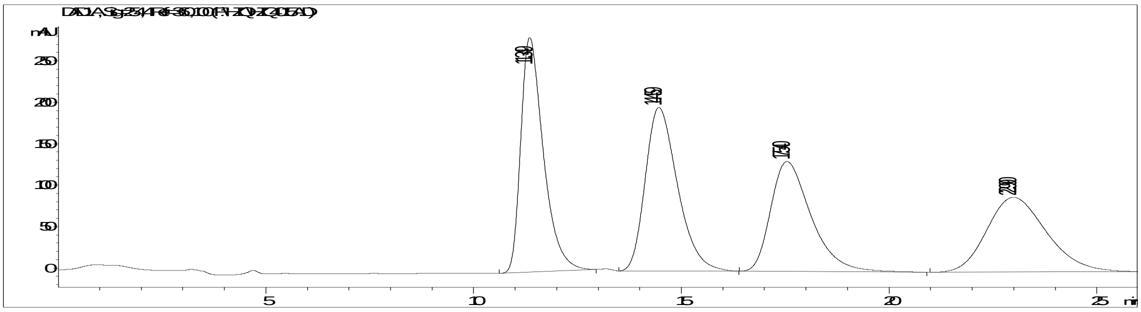

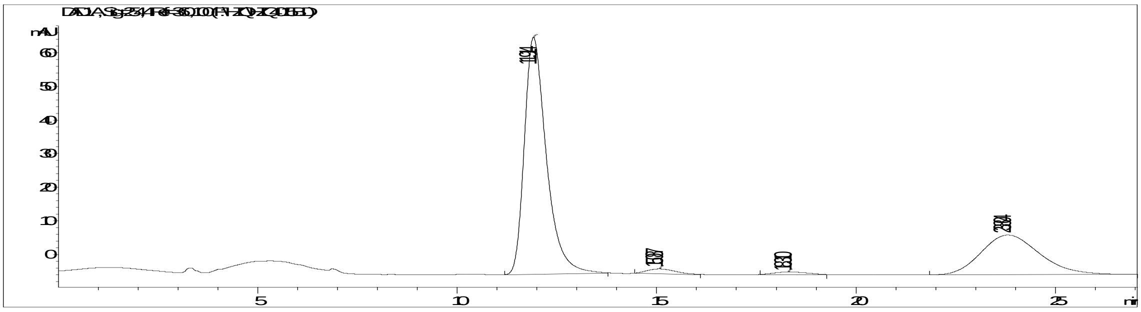

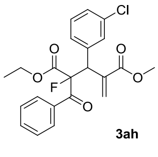

3ah

Colorless oil, 81% yield; 91% ee; 3:1 dr; ^{1}H NMR (400 MHz, $CDCl_3$) δ 8.03–7.79 (m, 2H), 7.63–7.27 (m, 5H), 7.17–7.10 (m, 2H), 6.56–5.94 (m, 2H), 5.54–5.30 (m, 1H), 4.37–4.04 (m, 2H), 3.72 (s, 3H), 1.26–1.11 (m, 3H). ^{13}C NMR (100 MHz, $CDCl_3$) δ 192.3, 192.0, 166.2, 165.9, 165.6, 138.0, 137.3, 134.4, 134.4, 134.2, 134.1, 133.9, 133.6, 130.5, 130.1, 129.9, 129.8, 129.6, 129.6, 129.3, 129.0, 128.6, 128.3, 128.14, 127.9, 127.8, 127.8, 102.0, 63.2, 52.4, 48.6, 48.4, 13.8. HRMS (ESI) *m*/*z* 419.1051 (M + H^+), calcd. for $C_{22}H_{21}ClFO_5$ 419.1056.

The ee was determined by HPLC analysis. Lux 5u cellulose-3 (250 x 4.6 mm); hexane/2-propanol = 70/30; flow rate 1.0 mL/min; 25 °C; 254 nm; retention time: 4.6 min (major) and 5.4 min (minor).

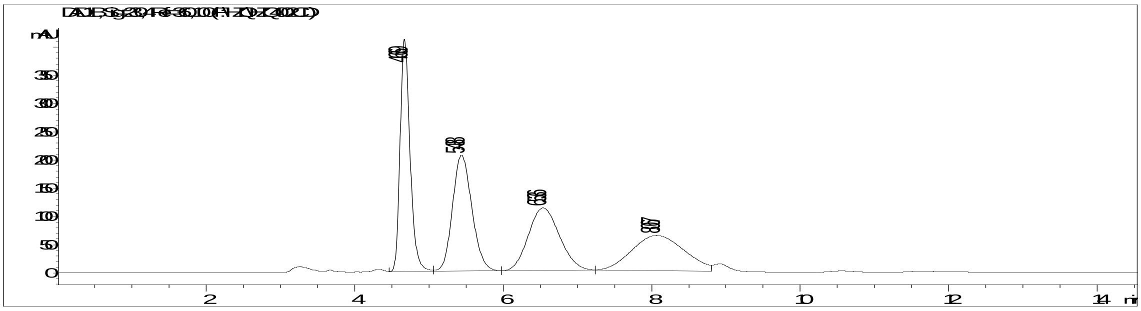

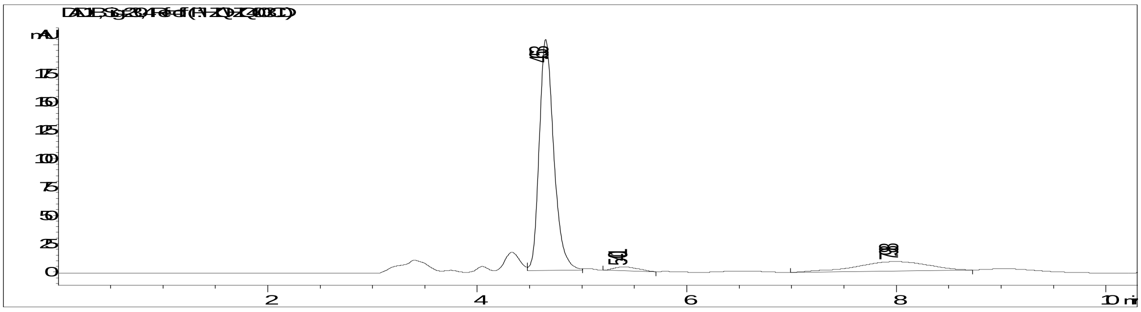

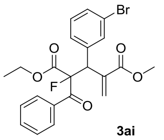

3ai

Colorless oil, 78% yield; 90% ee; 4:1 dr; ^{1}H NMR (400 MHz, $CDCl_3$) δ 8.07–7.78 (m, 2H), 7.65–7.43 (m, 2H), 7.37 (t, J = 8.5 Hz, 3H), 7.31–7.26 (m, 2H), 6.58–5.93 (m, 2H), 5.55–5.31 (m, 1H), 4.43–4.03 (m, 2H), 3.73 (s, 3H), 1.27–1.12 (m, 3H). ^{13}C NMR (100 MHz, $CDCl_3$) δ 192.3, 192.0, 190.7, 166.5, 166.3, 165.8, 165.6, 141.5, 139.1, 138.4, 135.1, 135.0, 133.4, 133.4, 132.9, 132.0, 131.6, 131.4, 131.3, 131.0, 131.0, 130.5, 130.5, 129.9, 129.6, 128.8, 128.4, 128.3, 128.2, 128.0, 127.6, 127.2, 127.2, 126.1, 124.8, 104.5, 102.4, 63.2, 60.4, 52.3, 51.6, 49.2, 49.0, 21.0, 14.2, 13.9, 13.8. HRMS (ESI) m/z 419.1051 (M + H$^+$), calcd. for $C_{22}H_{21}BrFO_5$ 419.1056.

The ee was determined by HPLC analysis. Lux 5u cellulose-3 (250 x 4.6 mm); hexane/2-propanol = 80/20; flow rate 1.0 mL/min; 25 °C; 254 nm; retention time: 5.3 min (major) and 6.3 min (minor).

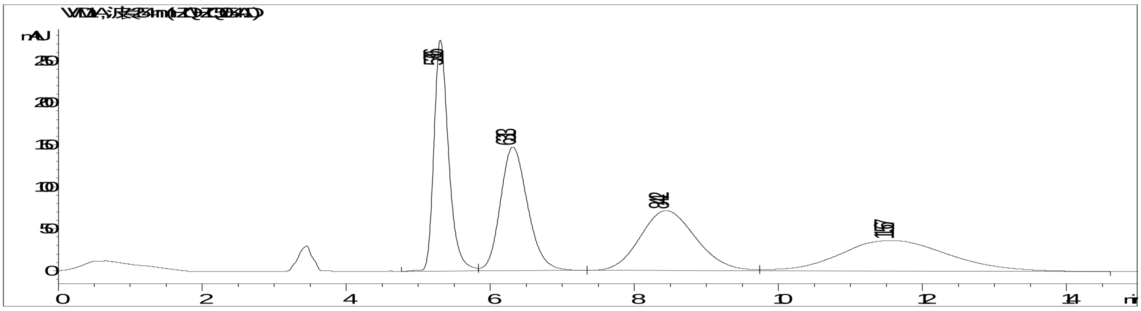

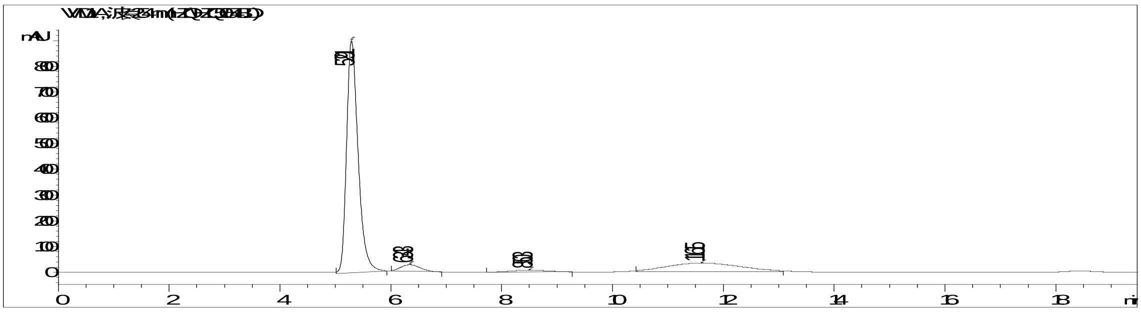

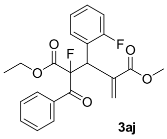

3aj

Colorless oil, 73% yield; 86% ee; 4:1 dr; ^{1}H NMR (400 MHz, $CDCl_3$) δ 8.02–7.78 (m, 2H), 7.63–7.31 (m, 4H), 7.21–6.94 (m, 3H), 6.57–5.93 (m, 2H),

5.83–5.73 (m, 1H), 4.42–4.05 (m, 2H), 3.73–3.70 (m, 3H), 1.25–1.11(m, 3H). ^{13}C NMR (100 MHz, $CDCl_3$) δ 191.9, 191.6, 166.2, 166.0, 165.7, 161.9, 159.4, 138.5, 137.6, 134.4, 134.0, 133.5, 131.7, 130.9, 129.8, 129.8, 129.6, 129.5, 129.3, 129.2, 129.0, 128.9, 128.6, 128.3, 126.9, 124.1, 124.0, 123.8, 123.8, 115.8, 115.6, 115.3, 103.5, 63.2, 52.3, 41.5, 41.35, 13.8, 13.5. HRMS (ESI) *m*/*z* 403.1364 (M + H$^+$), calcd. for $C_{22}H_{21}NF_2O_5$ 403.1352.

The ee was determined by HPLC analysis. Lux 5u cellulose-3 (250 x 4.6 mm); hexane/2-propanol = 70/30; flow rate 1.0 mL/min; 25 ^{o}C; 254 nm; retention time: 5.8 min (major) and 7.5 min (minor).

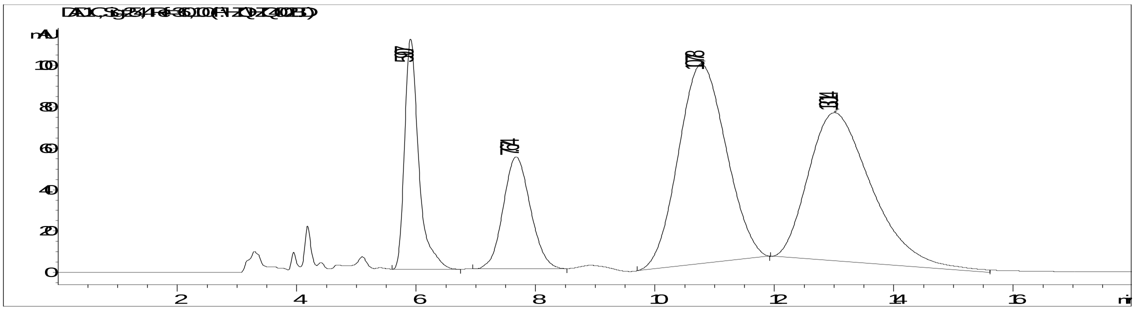

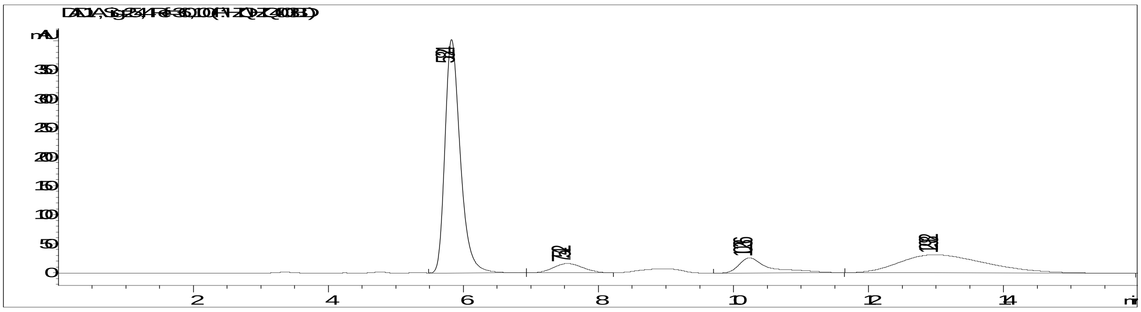

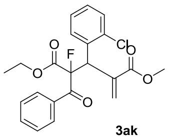

3ak

Colorless oil, 84% yield; 86% ee; 4:1 dr; ^{1}H NMR (400 MHz, $CDCl_3$) δ 8.03–7.79 (m, 2H), 7.63–7.27 (m, 5H), 7.17–7.10 (m, 2H), 6.56–5.94 (m, 2H), 5.54–5.30 (m, 1H), 4.37–4.04 (m, 2H), 3.72 (s, 3H), 1.26–1.11 (m, 3H). ^{13}C NMR (100 MHz, $CDCl_3$) δ 192.4, 192.1, 166.2, 165.9, 165.6, 138.2, 134.4, 134.1, 133.8, 133.6, 133.5, 132.0 (two peaks), 131.3, 129.9, 129.8, 129.6, 129.6, 128.6, 128.6, 128.3, 127.5, 127.4, 104.2, 102.1, 63.1, 52.3, 48.4, 48.2, 13.8. HRMS (ESI) *m*/*z* 419.1053 (M + H$^+$), calcd. for $C_{22}H_{21}ClFO_5$ 419.1056.

The ee was determined by HPLC analysis. CHIRALPAK IB (4.6 mm i.d. x 250 mm); hexane/2-propanol = 95/05; flow rate 0.5 mL/min; 25 °C; 254 nm; retention time: 11.4 min (major) and 13.2 min (minor).

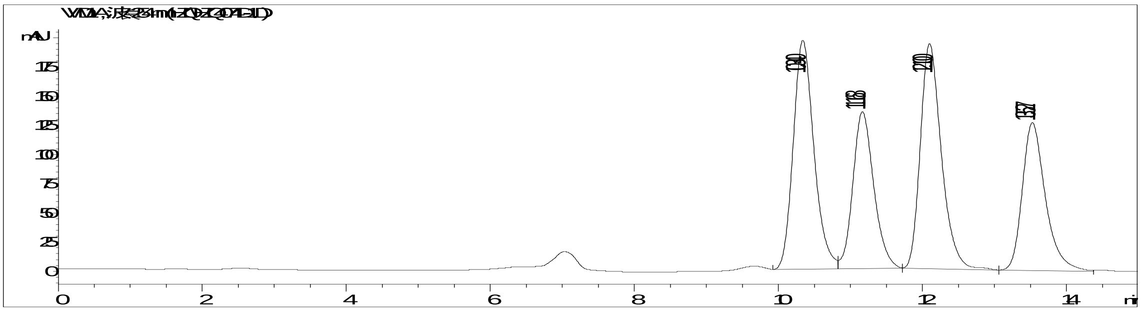

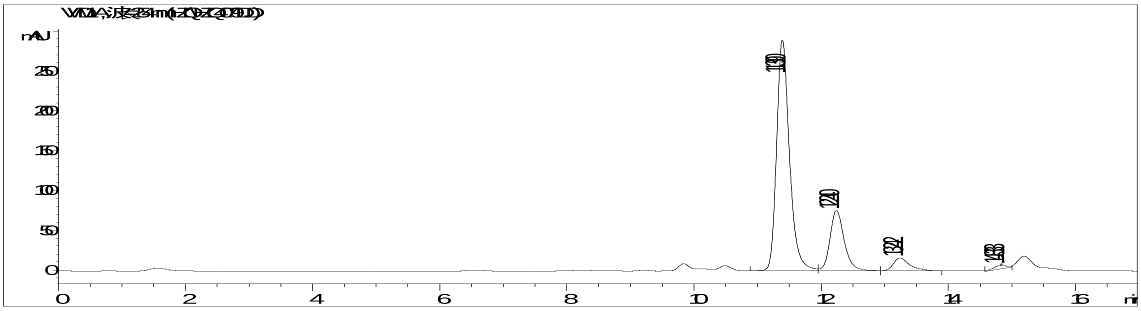

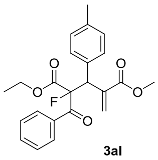

3al

Colorless oil, 53% yield; 91% ee; 4:1 dr; ^{1}H NMR (400 MHz, $CDCl_3$) δ 8.05–7.71 (m, 2H), 7.61–7.26 (m, 3H), 7.26–7.23 (m, 2H), 7.12–7.00 (m, 2H), 6.50–5.93 (m, 2H), 5.52–5.28 (m, 1H), 4.32–4.08 (m, 2H), 3.70 (s, 3H), 2.31–2.23 (m, 3H), 1.26–1.10 (m, 3H). ^{13}C NMR (100 MHz, $CDCl_3$) δ 192.9, 192.6, 166.4, 166.2, 165.8, 138.6, 137.5, 137.1, 134.8, 134.7, 133.9, 133.2, 132.1, 130.4, 130.4, 129.8, 129.8, 129.6, 129.5, 129.1, 128.9, 128.6, 128.1, 127.1, 127.0, 125.9, 104.5, 102.4, 62.7, 52.2, 48.9, 48.7, 48.55, 21.0, 13.8. HRMS (ESI) *m/z* 399.1611 (M + H^+), calcd. for $C_{23}H_{24}FO_5$ 399.1602.

The ee was determined by HPLC analysis. Lux 5u cellulose-3 (250 x 4.6 mm); hexane/2-propanol = 70/30; flow rate 1.0 mL/min; 25 °C; 254 nm; retention time: 4.5 min (major) and 5.5 min (minor).

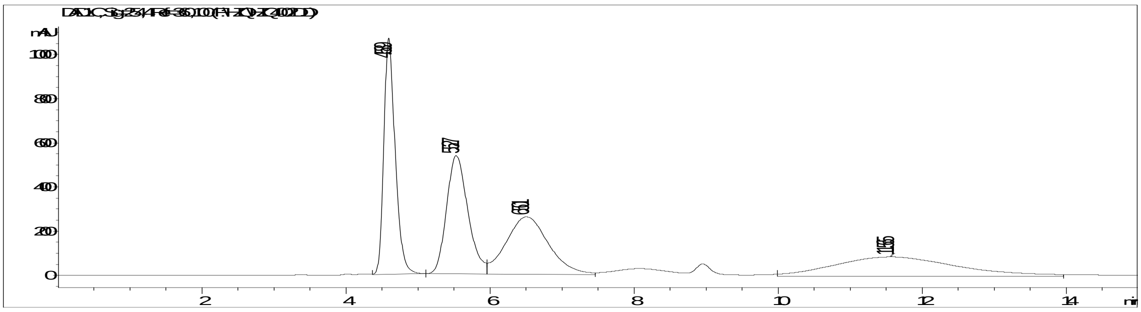

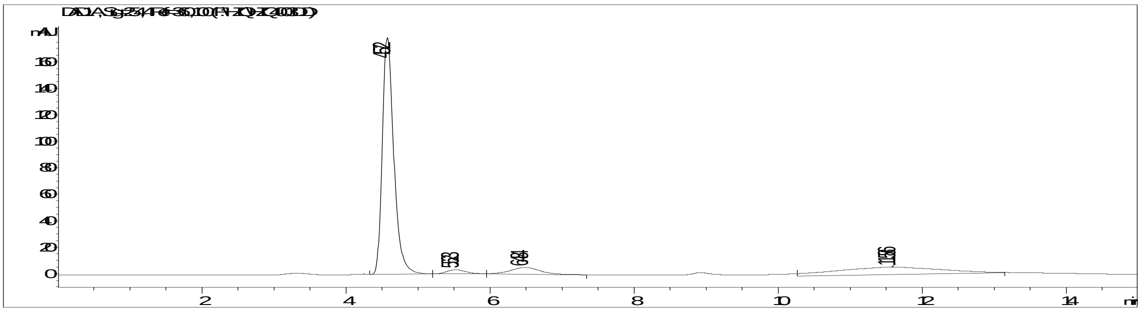

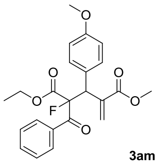

3am

Colorless oil, 50% yield; 91% ee; 3:1 dr; ^{1}H NMR (400 MHz, $CDCl_3$) δ 8.05–7.71 (m, 2H), 7.62–7.28 (m, 3H), 7.26–6.98 (m, 4H), 6.51–5.92 (m, 2H), 5.52–5.28 (m, 1H), 4.38–4.03 (m, 2H), 3.70 (d, J = 1.7 Hz, 3H), 2.31–2.23 (m, 3H), 1.25–1.10 (m, 3H). ^{13}C NMR (100 MHz, $CDCl_3$) δ 192.9, 192.6, 191.7, 191.4, 166.4, 166.2, 165.9, 139.3, 138.7, 137.6, 137.1, 134.8, 134.7, 134.4, 133.9, 133.2, 132.1, 132.1, 130.4, 130.4, 129.8, 129.8, 129.6, 129.5, 129.1, 128.9, 128.6, 128.1, 127.1, 127.1, 125.9, 125.8, 104.5, 102.4, 63.0, 62.9, 52.2, 48.7, 48.6, 21.0, 13.8. HRMS (ESI) m/z 437.1380 (M + Na^+), calcd. for $C_{23}H_{23}FO_5Na$ 437.1371.

The ee was determined by HPLC analysis. Lux 5u cellulose-3 (250 x 4.6 mm); hexane/2-propanol = 95/05; flow rate 0.5 mL/min; 25 °C; 254 nm; retention time: 5.4 min (major) and 6.1 min (minor).

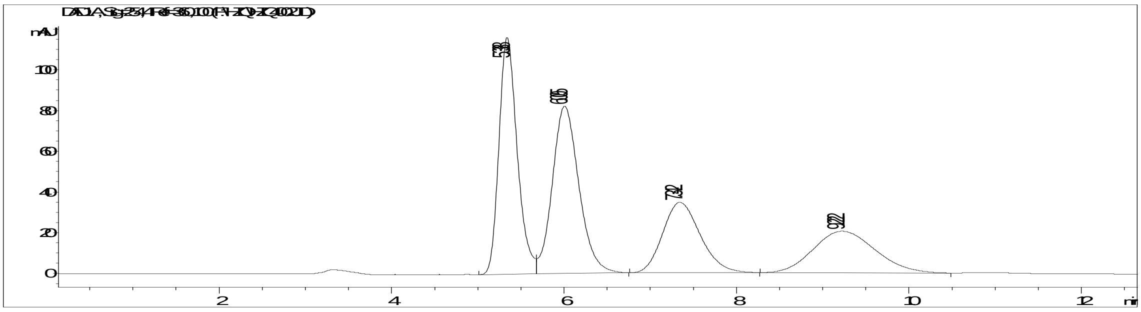

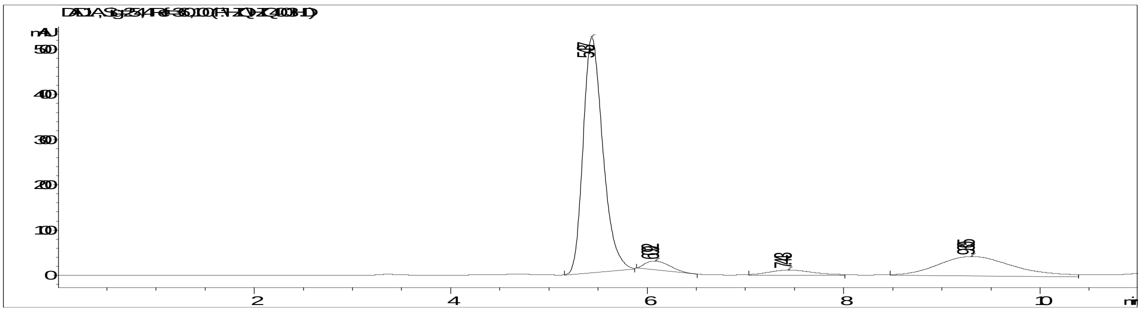

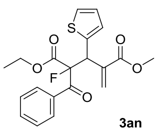

3an

Colorless oil, 78% yield; 92% ee; 4:1 dr; ^{1}H NMR (400 MHz, $CDCl_3$) δ 8.07–7.83 (m, 2H), 7.63–7.33 (m, 3H), 7.25–6.81 (m, 3H), 6.56–5.99 (m, 2H), 5.91–5.67 (m,

1H), 4.35–4.10 (m, 2H), 3.78–3.76 (m, 3H), 1.23–1.14 (m, 3H). ^{13}C NMR (100 MHz, $CDCl_3$) δ 192.3, 192.0, 166.2, 165.6, 165.4, 138.2, 137.9, 134.5, 134.5, 134.1, 133.6, 129.9, 129.9, 129.7, 129.7, 128.6, 128.3, 128.3, 128.2, 128.0, 126.6, 126.5, 126.0, 125.7, 104.0, 101.9, 76.7, 63.1, 52.4, 44.1, 43.9, 13.8. HRMS (ESI) *m*/*z* 413.0832 (M + Na^+), calcd. for $C_{20}H_{19}FO_5SNa$ 413.0829.

The ee was determined by HPLC analysis. Lux 5u cellulose-3 (250 x 4.6 mm); hexane/2-propanol = 80/20; flow rate 1.0 mL/min; 25 °C; 254 nm; retention time: 6.8 min (major) and 10.7 min (minor).

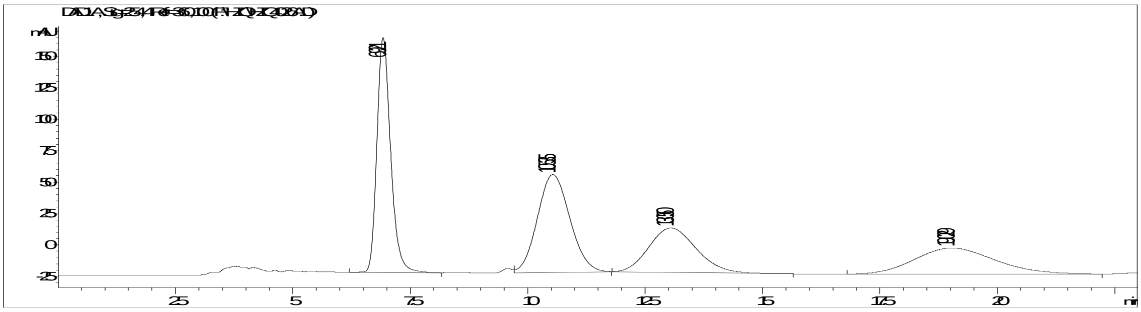

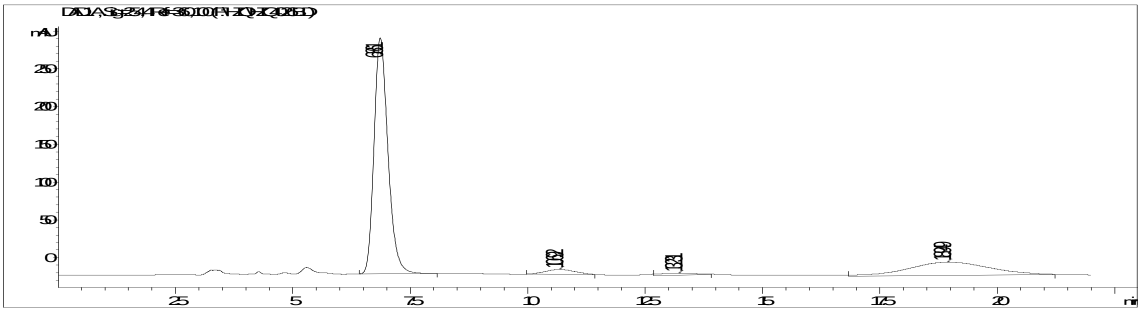

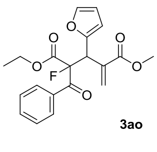

3ao

Colorless oil, 73% yield; 84% ee; 3:1 dr; ^{1}H NMR (400 MHz, $CDCl_3$) δ 8.03–7.79 (m, 2H), 7.63–7.32 (m, 4H), 7.27–7.23 (m, 1H), 7.16–7.13 (m, 1H), 6.56–5.94 (m, 2H), 5.54–5.30 (m, 1H), 4.37–4.04 (m, 2H), 3.72 (s, 3H), 1.26–1.11 (m, 3H). ^{13}C NMR (100 MHz, $CDCl_3$) δ 192.2, 191.9, 166.6, 166.3, 165.5, 165.2, 150.3, 149.8, 145.9, 142.8, 142.4, 136.6, 135.5, 134.4, 134.4, 134.0, 133.5, 130.4, 130.4, 129.9, 129.8, 129.7, 129.6, 128.6, 128.3, 110.4, 110.3, 109.5, 109.4, 103.1, 101.0, 63.1, 63.0, 61.5, 52.4, 52.4, 42.9, 42.7, 13.8, 13.7. HRMS (ESI) *m*/*z* 397.1063 (M + Na^+), calcd. for $C_{20}H_{19}FO_6Na$ 397.1058.

The ee was determined by HPLC analysis. Lux 5u cellulose-3 (250 x 4.6 mm); hexane/2-propanol = 90/10; flow rate 1.0 mL/min; 25 °C; 254 nm; retention time: 11.3 min (major) and 12.2 min (minor).

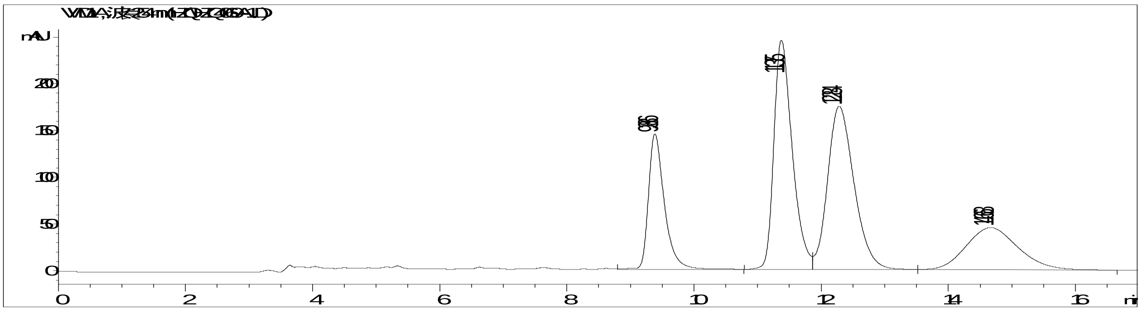

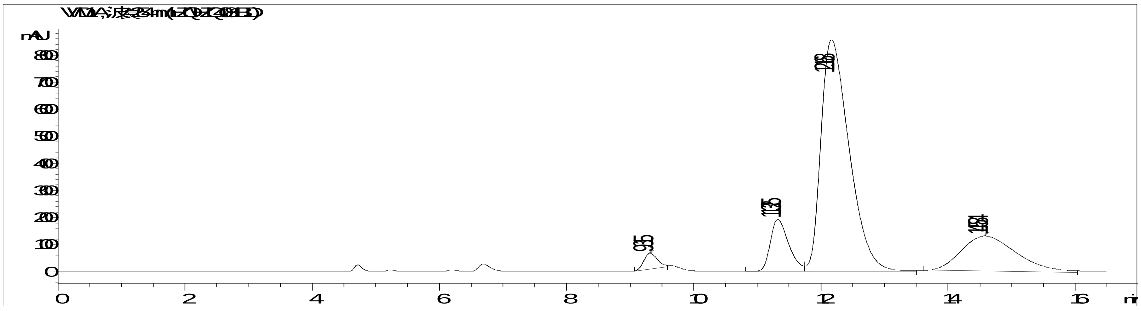

4 Copies of NMR Spectra

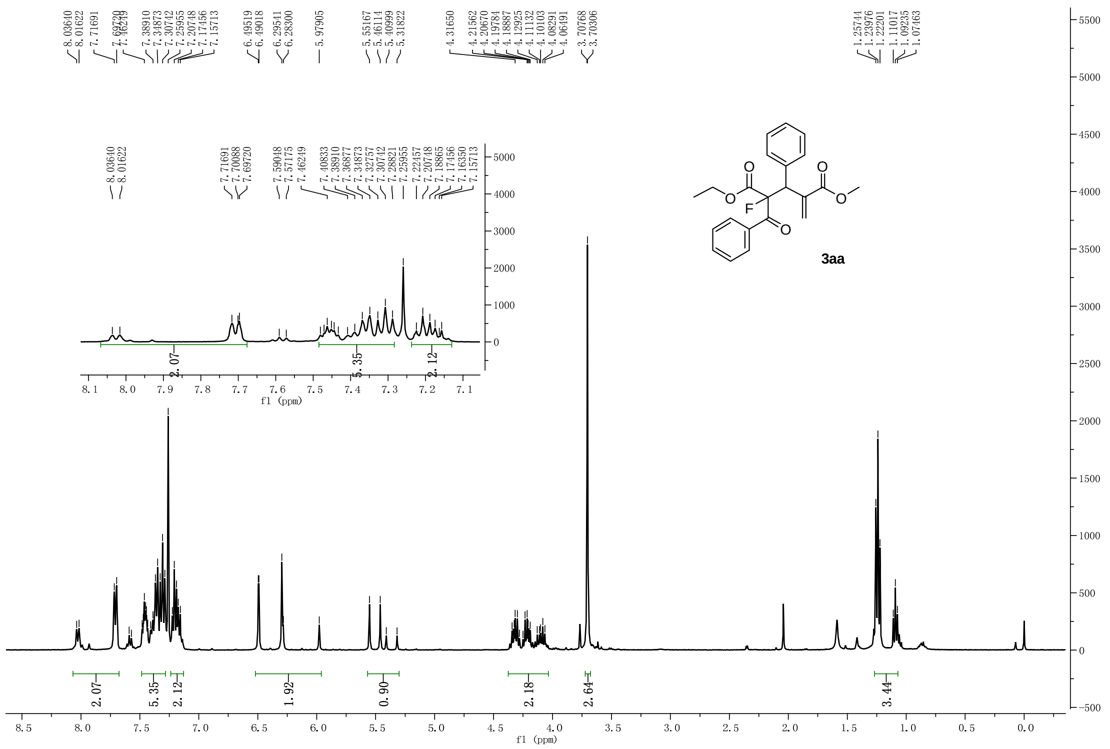

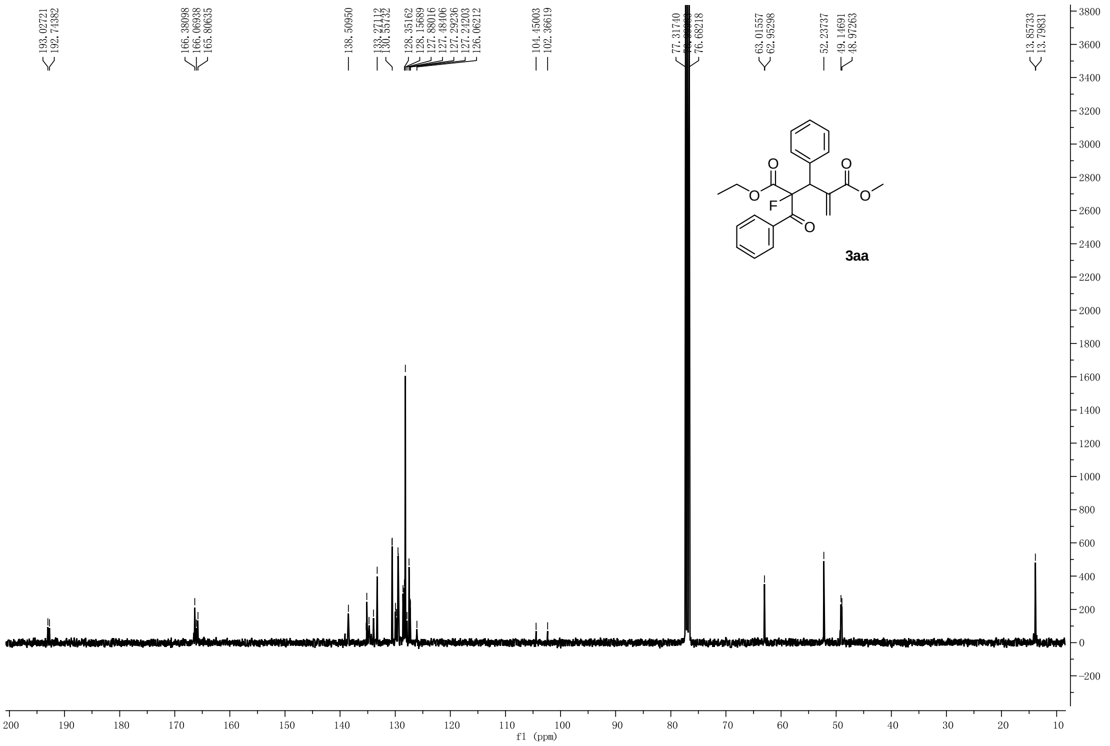

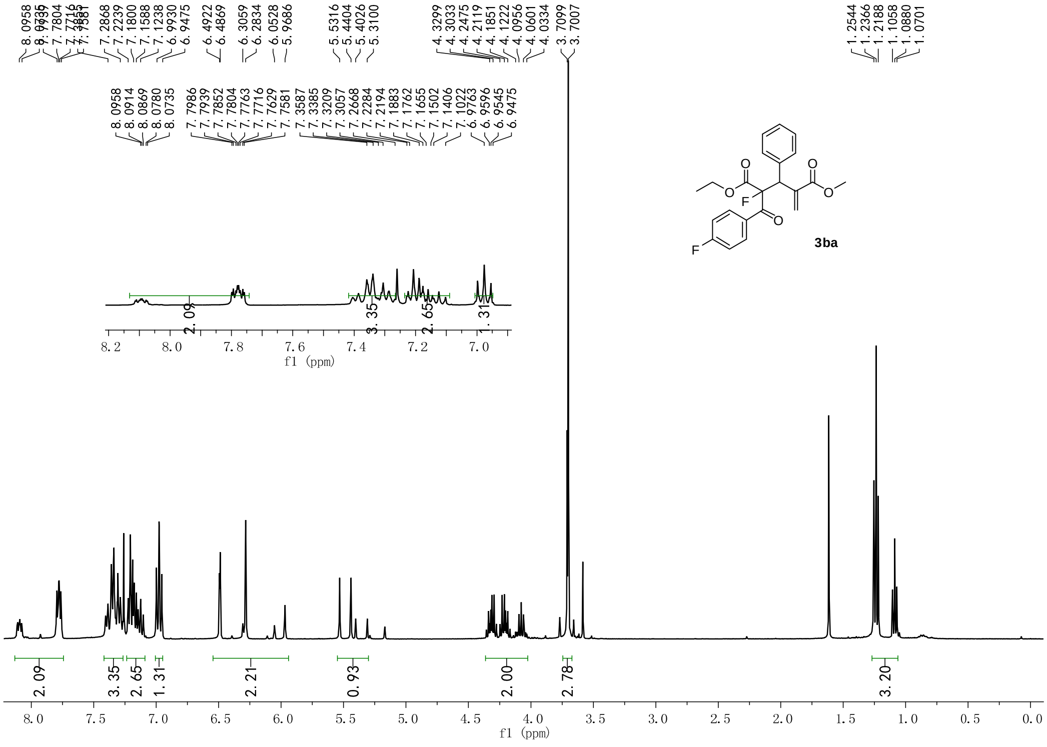
8.0958
7.7804
7.2868
7.2239
7.1800
7.1588
7.1238
6.9930
6.9475
6.4922
6.4869
6.3059
6.2834
6.0528
5.9686
5.5316
5.4404
5.4026
5.3100
4.3299
4.3033
4.2475
4.2119
4.1851
4.1222
4.0956
4.0601
4.0334
3.7099
3.7007
1.2544
1.2366
1.2188
1.1058
1.0880
1.0701
8.0958
8.0914
8.0869
8.0780
8.0735
7.7986
7.7939
7.7852
7.7804
7.7763
7.7716
7.7629
7.7581
7.3587
7.3385
7.3209
7.3057
7.2668
7.2284
7.2194
7.1883
7.1762
7.1655
7.1502
7.1406
7.1022
6.9763
6.9596
6.9545
6.9475
3ba
2.09
3.35
2.65
1.31
2.21
0.93
2.00
2.78
3.20
f1 (ppm)

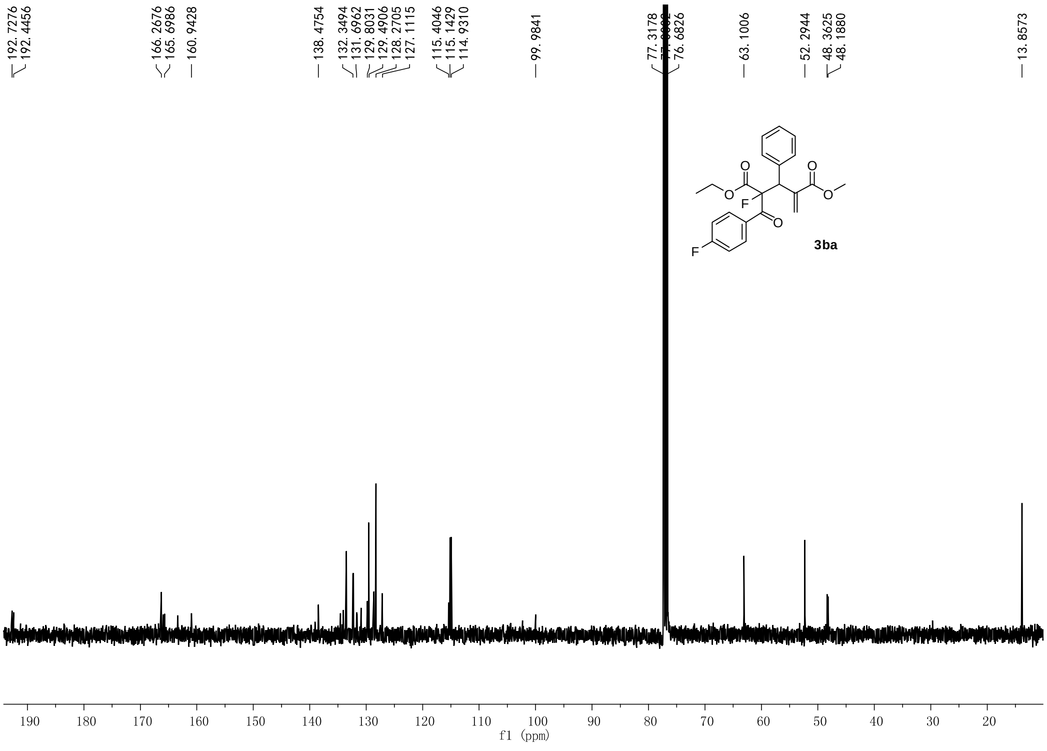
192.7276
192.4456
166.2676
165.6986
160.9428
138.4754
132.3494
131.6962
129.8031
129.4906
128.2705
127.1115
115.4046
115.1429
114.9310
99.9841
77.3178
76.6826
63.1006
52.2944
48.3625
48.1880
13.8573
3ba
f1 (ppm)

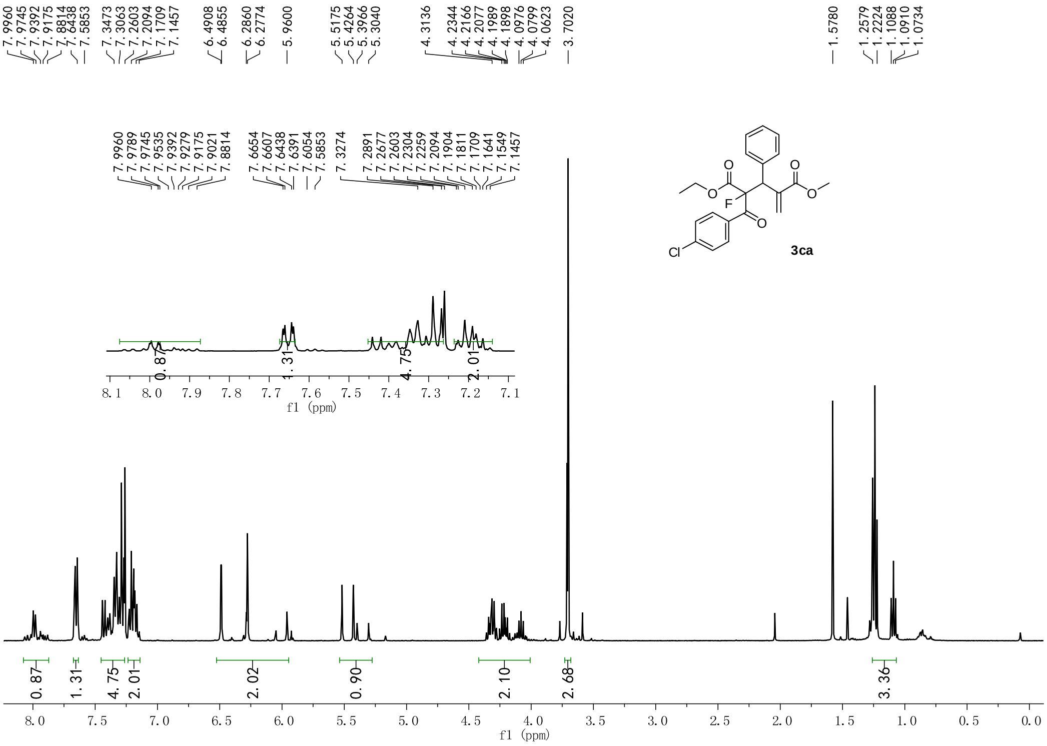
7.9960
7.9745
7.9392
7.9175
7.8814
7.6438
7.5853
7.3473
7.3063
7.2603
7.2094
7.1709
7.1457
6.4908
6.4855
6.2860
6.2774
5.9600
5.5175
5.4264
5.3966
5.3040
4.3136
4.2344
4.2166
4.2077
4.1989
4.1898
4.0976
4.0799
4.0623
3.7020
1.5780
1.2579
1.2224
1.1088
1.0910
1.0734
3ca
0.87
1.31
4.75
2.01
2.02
0.90
2.10
2.68
3.36
f1 (ppm)

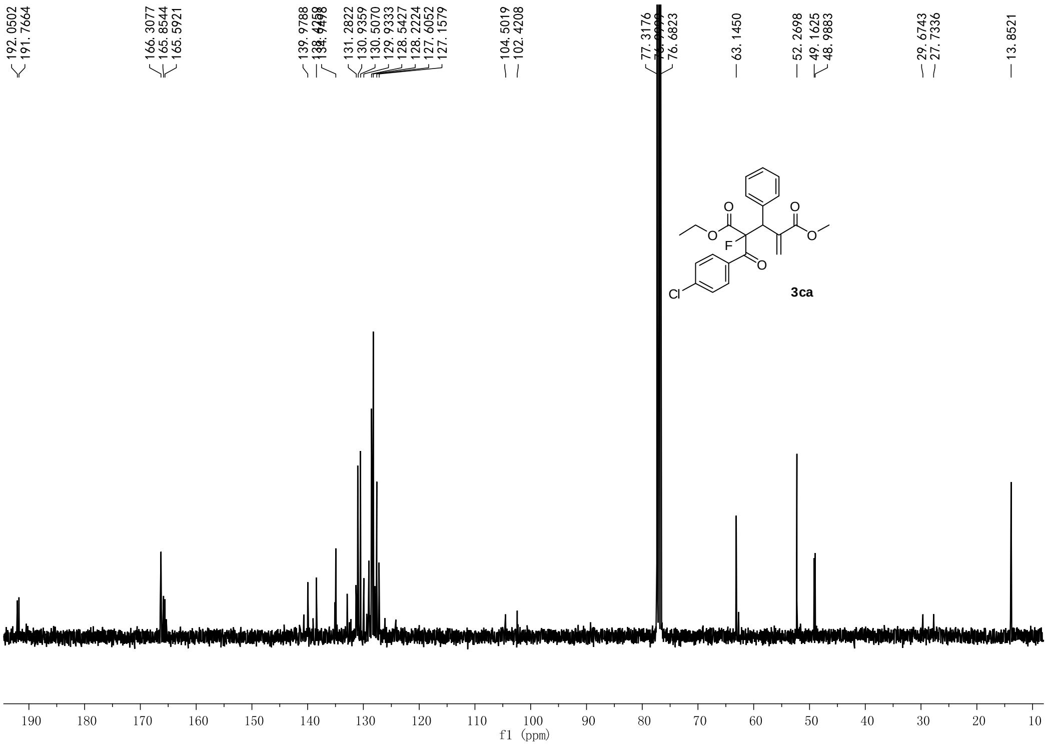
192.0502
191.7664
166.3077
165.8544
165.5921
139.9788
131.2822
130.9359
130.5070
129.9333
128.5427
128.2224
127.6052
127.1579
104.5019
102.4208
77.3176
76.6823
63.1450
52.2698
49.1625
48.9883
29.6743
27.7336
13.8521
3ca
f1 (ppm)

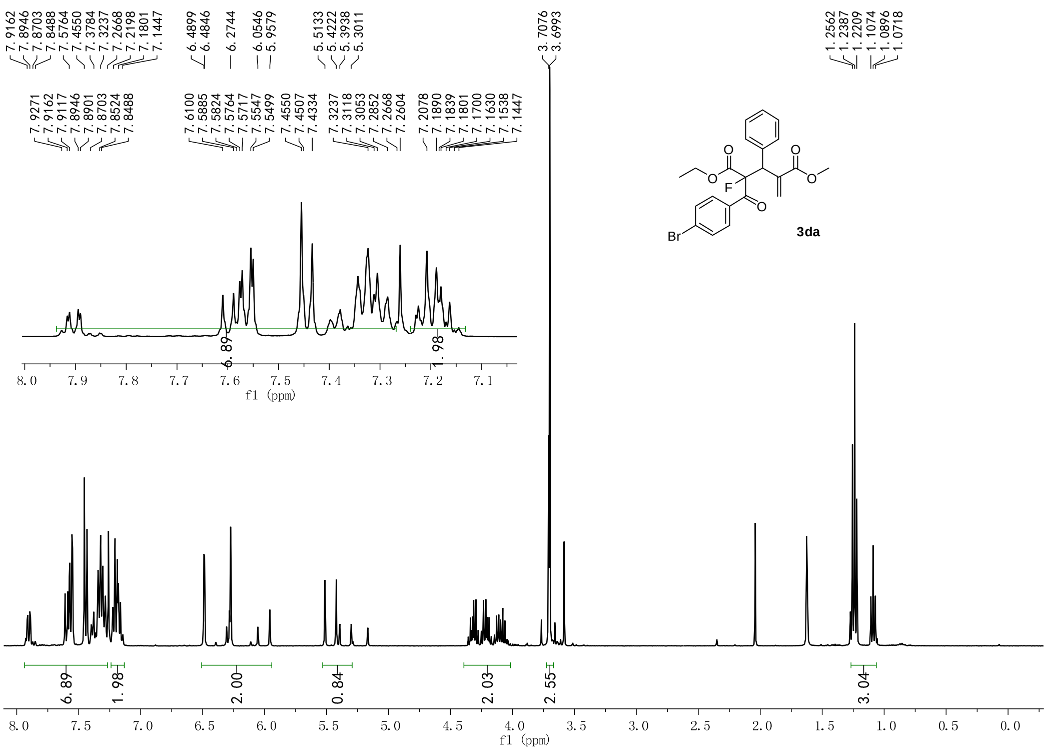
3da
Br
F
f1 (ppm)

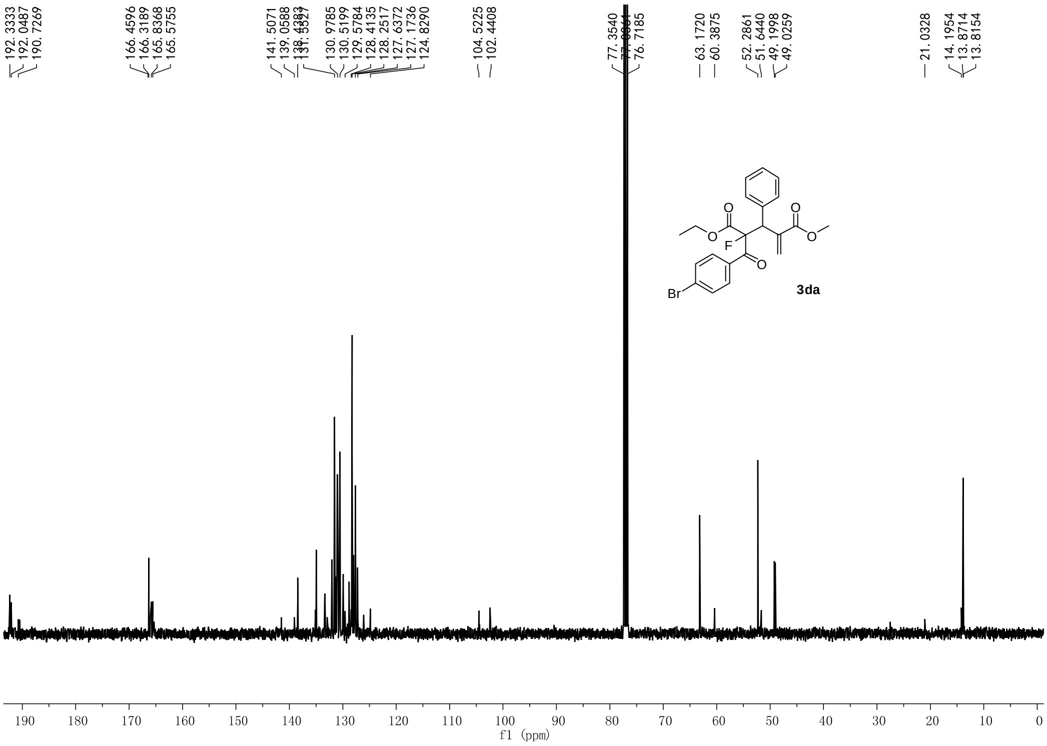
3da
Br
F
f1 (ppm)

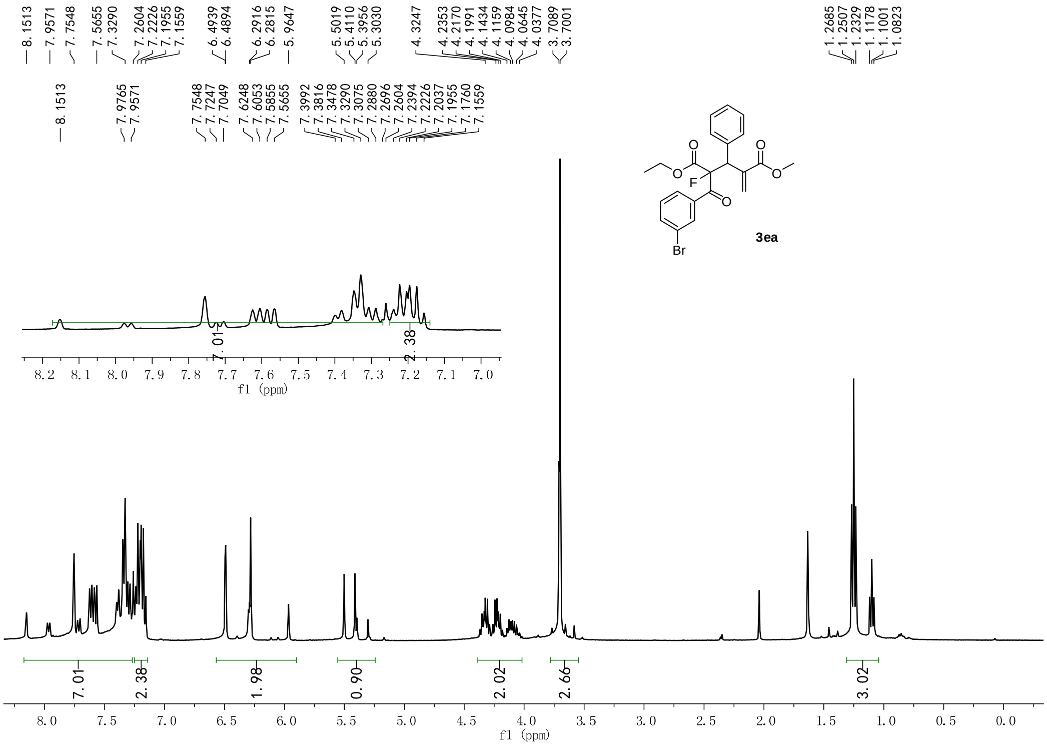
8.1513
7.9571
7.7548
7.5655
7.3290
7.2604
7.2226
7.1955
7.1559
6.4939
6.4894
6.2916
6.2815
5.9647
5.5019
5.4110
5.3956
5.3030
4.3247
4.2353
4.2170
4.1991
4.1434
4.1159
4.0984
4.0645
4.0377
3.7089
3.7001
1.2685
1.2507
1.2329
1.1178
1.1001
1.0823
8.1513
7.9765
7.9571
7.7548
7.7247
7.7049
7.6248
7.6053
7.5855
7.5655
7.3992
7.3816
7.3478
7.3290
7.3075
7.2880
7.2696
7.2604
7.2394
7.2226
7.2037
7.1955
7.1760
7.1559
O
F
O
O
O
O
Br
3ea
7.01
2.38
8.2 8.1 8.0 7.9 7.8 7.7 7.6 7.5 7.4 7.3 7.2 7.1 7.0
f1 (ppm)
7.01
2.38
1.98
0.90
2.02
2.66
3.02
8.0 7.5 7.0 6.5 6.0 5.5 5.0 4.5 4.0 3.5 3.0 2.5 2.0 1.5 1.0 0.5 0.0
f1 (ppm)

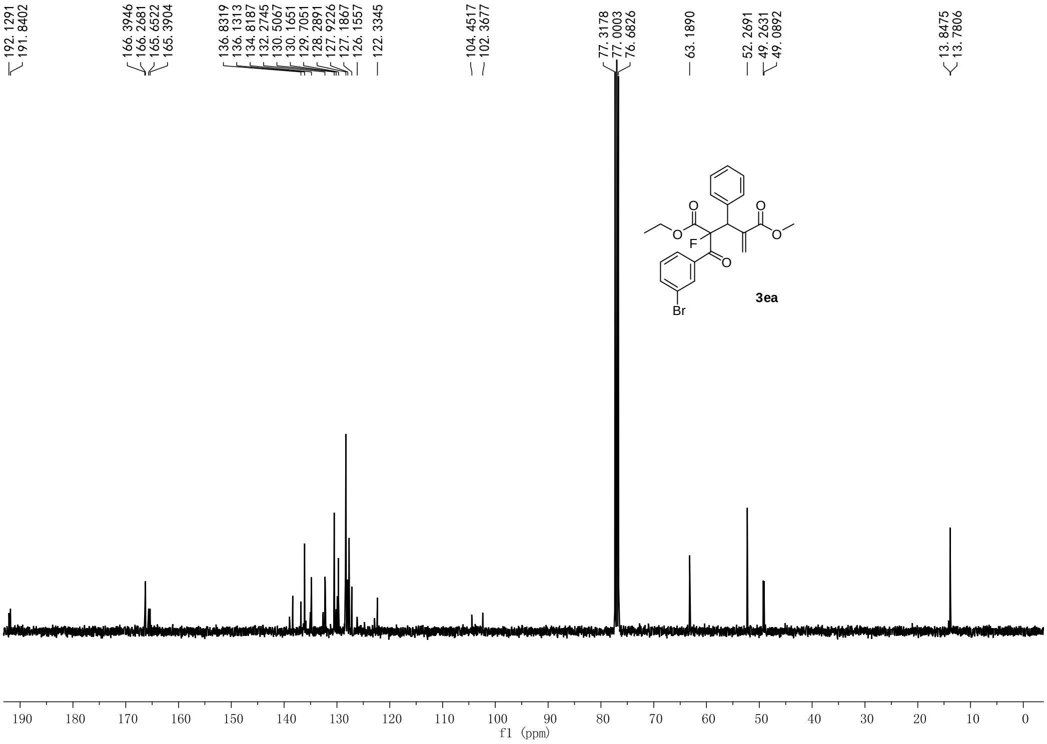
192.1291
191.8402
166.3946
166.2681
165.6522
165.3904
136.8319
136.1313
134.8187
132.2745
130.5067
130.1651
129.7051
128.2891
127.9226
127.1867
126.1557
122.3345
104.4517
102.3677
77.3178
77.0003
76.6826
63.1890
52.2691
49.2631
49.0892
13.8475
13.7806
O
F
O
O
O
O
Br
3ea
190 180 170 160 150 140 130 120 110 100 90 80 70 60 50 40 30 20 10 0
f1 (ppm)

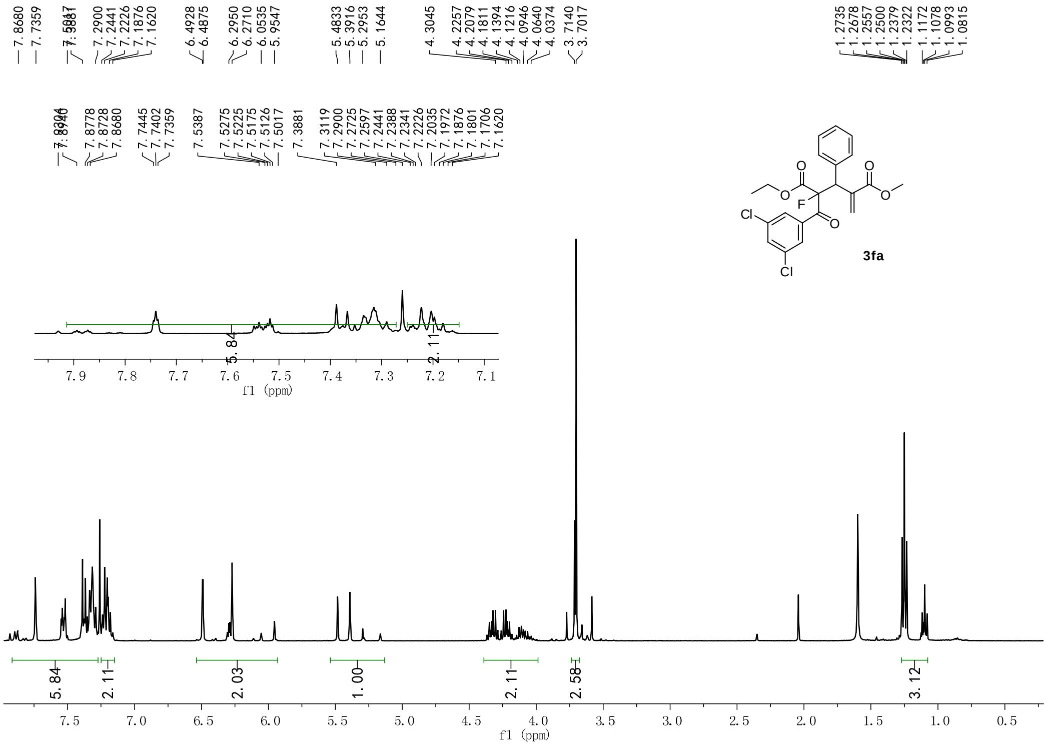
7.8680
7.7359
7.2900
7.2441
7.2226
7.1876
7.1620
6.4928
6.4875
6.2950
6.2710
6.0535
5.9547
5.4833
5.3916
5.2953
5.1644
4.3045
4.2257
4.2079
4.1811
4.1394
4.1216
4.0946
4.0640
4.0374
3.7140
3.7017
1.2735
1.2678
1.2557
1.2500
1.2379
1.2322
1.1172
1.1078
1.0993
1.0815
7.8778
7.8728
7.8680
7.7445
7.7402
7.7359
7.5387
7.5275
7.5225
7.5175
7.5126
7.5017
7.3881
7.3119
7.2900
7.2725
7.2597
7.2441
7.2388
7.2341
7.2226
7.2035
7.1972
7.1876
7.1801
7.1706
7.1620
Cl
Cl
F
O
3fa
5.84
2.11
2.03
1.00
2.58
3.12
f1 (ppm)

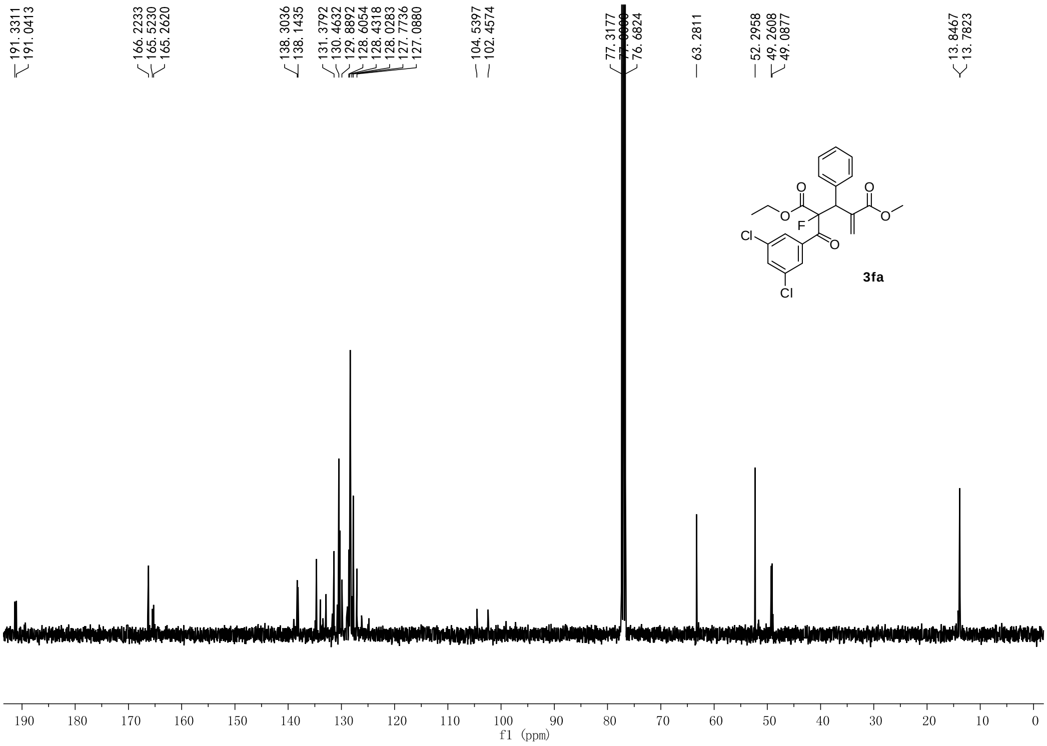
191.3311
191.0413
166.2233
165.5230
165.2620
138.3036
138.1435
131.3792
130.4632
129.8892
128.6054
128.4318
128.0283
127.7736
127.0880
104.5397
102.4574
77.3177
76.6824
63.2811
52.2958
49.2608
49.0877
13.8467
13.7823
Cl
Cl
F
O
3fa
f1 (ppm)

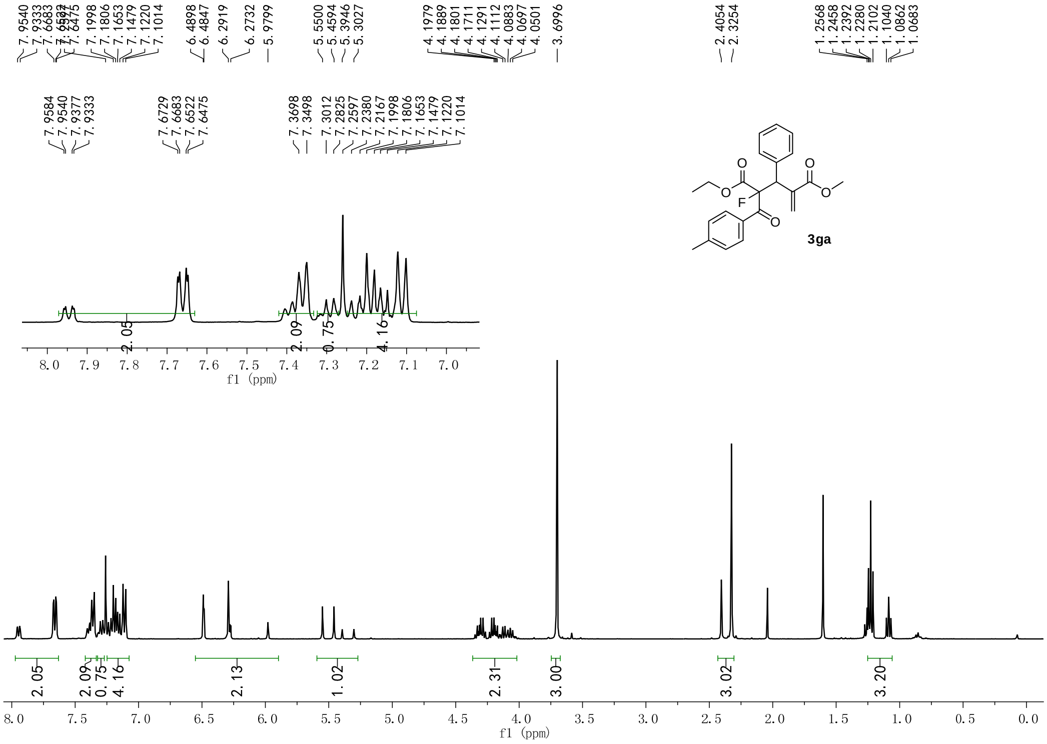
3ga
f1 (ppm)

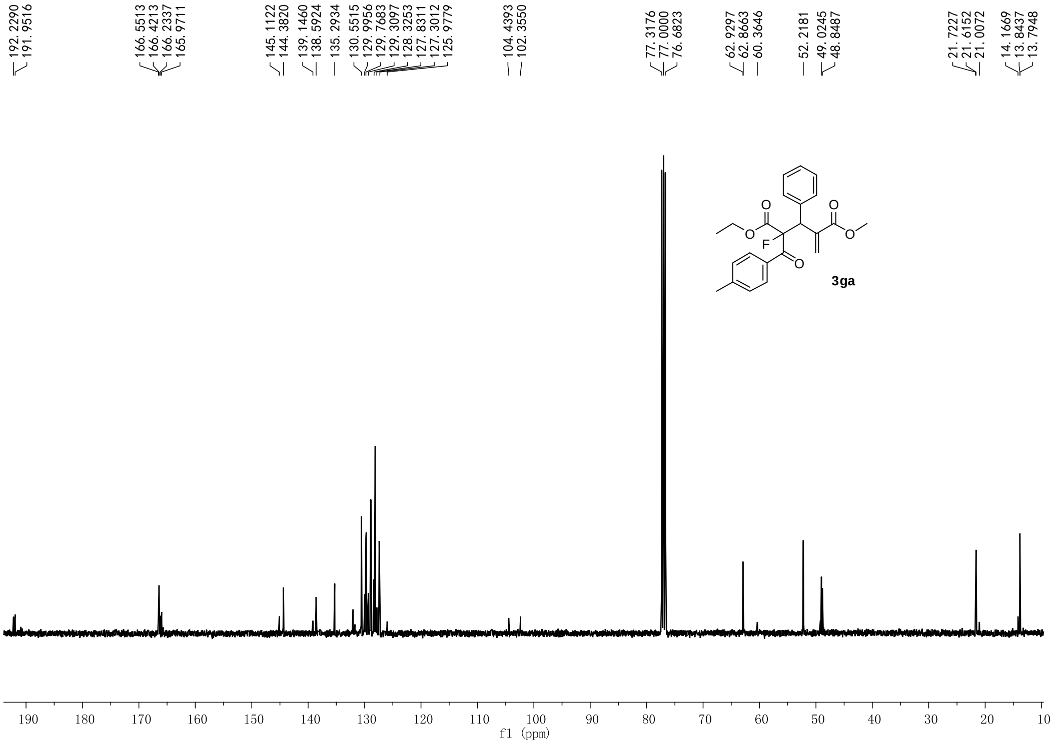
3ga
f1 (ppm)

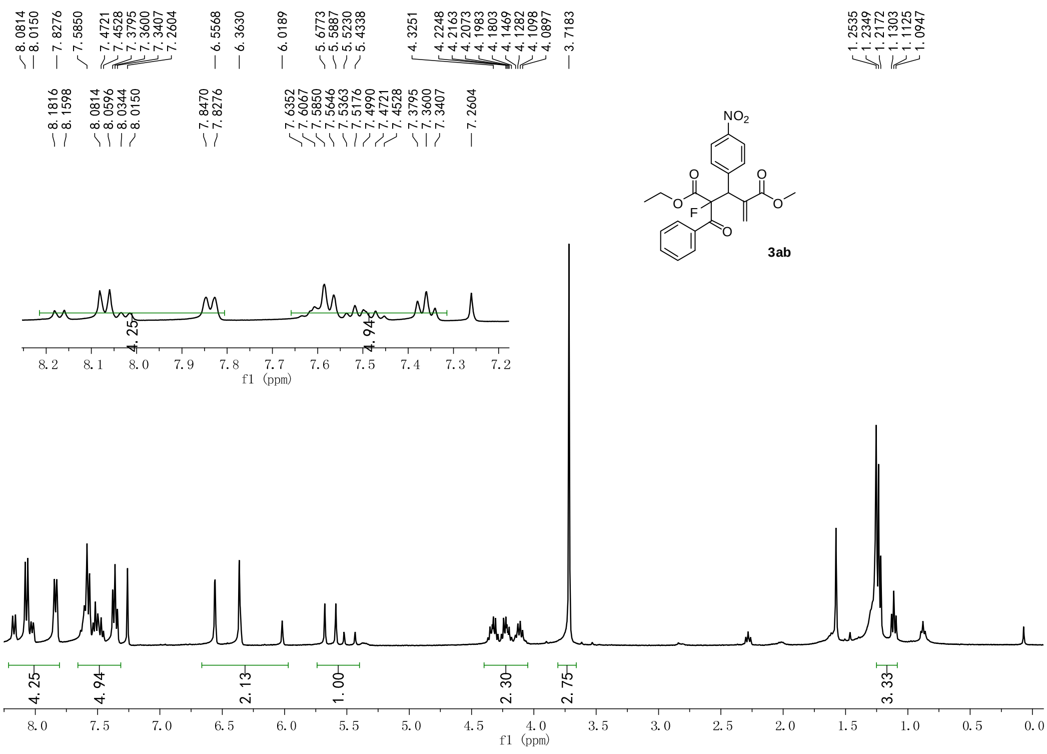
8.0814
8.0150
7.8276
7.5850
7.4721
7.4528
7.3795
7.3600
7.3407
7.2604
6.5568
6.3630
6.0189
5.6773
5.5887
5.5230
5.4338
4.3251
4.2248
4.2163
4.2073
4.1983
4.1803
4.1469
4.1282
4.1098
4.0897
3.7183
1.2535
1.2349
1.2172
1.1303
1.1125
1.0947
8.1816
8.1598
8.0814
8.0596
8.0344
8.0150
7.8470
7.8276
7.6352
7.6067
7.5850
7.5646
7.5363
7.5176
7.4990
7.4721
7.4528
7.3795
7.3600
7.3407
7.2604
NO2
F
O
3ab
4.25
4.94
f1 (ppm)
2.13
1.00
2.30
2.75
3.33

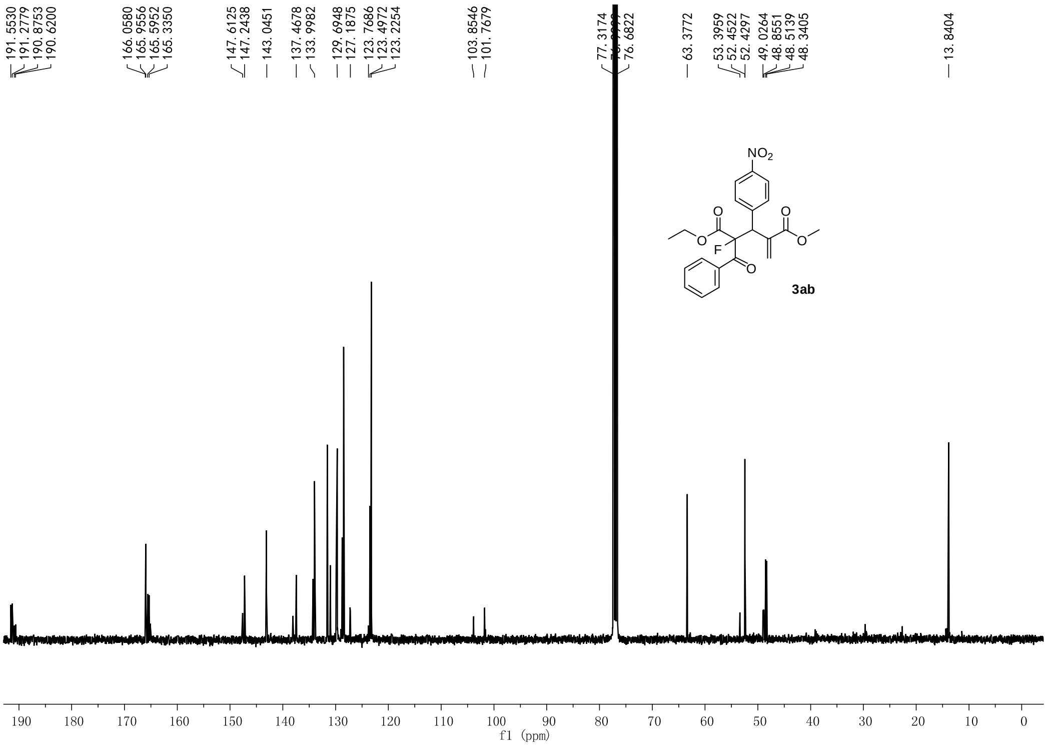
191.5530
191.2779
190.8753
190.6200
166.0580
165.9556
165.5952
165.3350
147.6125
147.2438
143.0451
137.4678
133.9982
129.6948
127.1875
123.7686
123.4972
123.2254
103.8546
101.7679
77.3174
[illegible]
76.6822
63.3772
53.3959
52.4522
52.4297
49.0264
48.8551
48.5139
48.3405
13.8404
NO2
F
O
3ab
f1 (ppm)

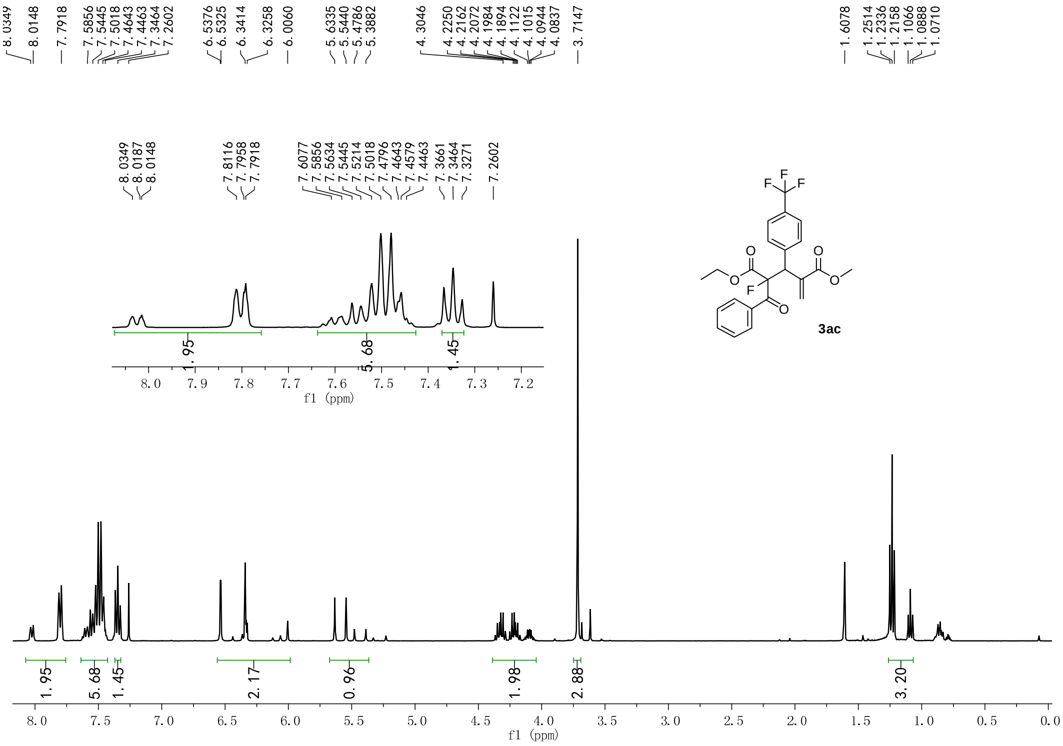

8.0349 8.0148 7.7918 7.5856 7.5445 7.5018 7.4643 7.4463 7.3464 7.2602 6.5376 6.5325 6.3414 6.3258 6.0060 5.6335 5.5440 5.4786 5.3882 4.3046 4.2250 4.2162 4.2072 4.1984 4.1894 4.1122 4.1015 4.0944 4.0837 3.7147 1.6078 1.2514 1.2336 1.2158 1.1066 1.0888 1.0710
8.0349 8.0187 8.0148 7.8116 7.7958 7.7918 7.6077 7.5856 7.5634 7.5445 7.5214 7.5018 7.4796 7.4643 7.4579 7.4463 7.3661 7.3464 7.3271 7.2602
3ac
f1 (ppm)

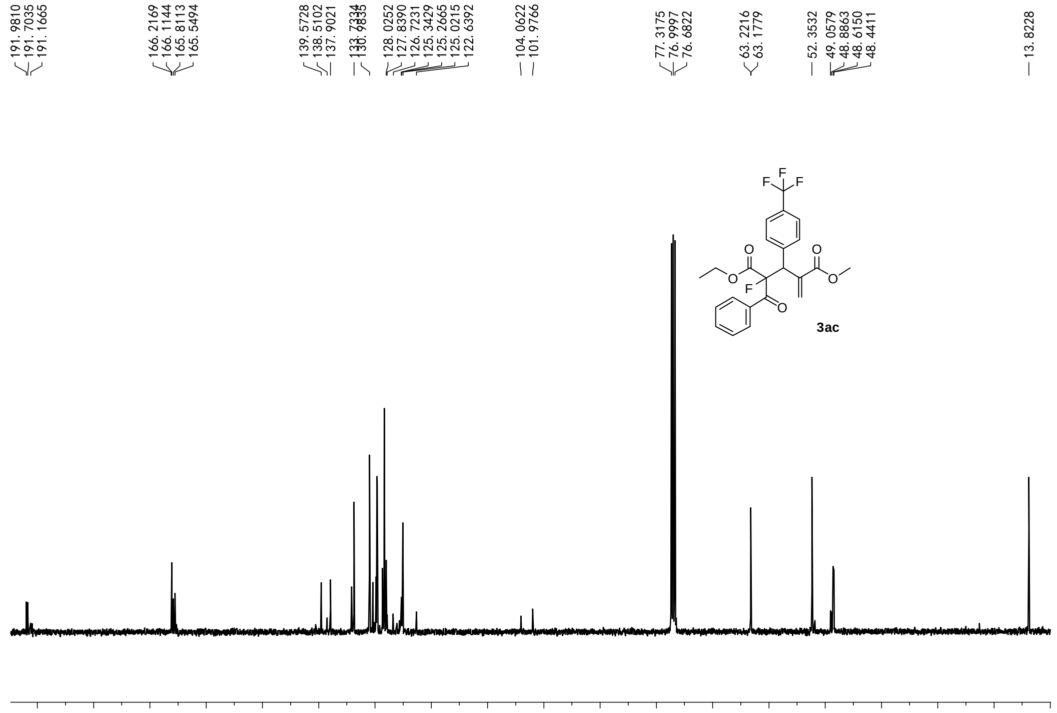

191.9810 191.7035 191.1665 166.2169 166.1144 165.8113 165.5494 139.5728 138.5102 137.9021 128.0252 127.8390 126.7231 125.3429 125.2665 125.0215 122.6392 104.0622 101.9766 77.3175 76.9997 76.6822 63.2216 63.1779 52.3532 49.0579 48.8863 48.6150 48.4411 13.8228
3ac
f1 (ppm)

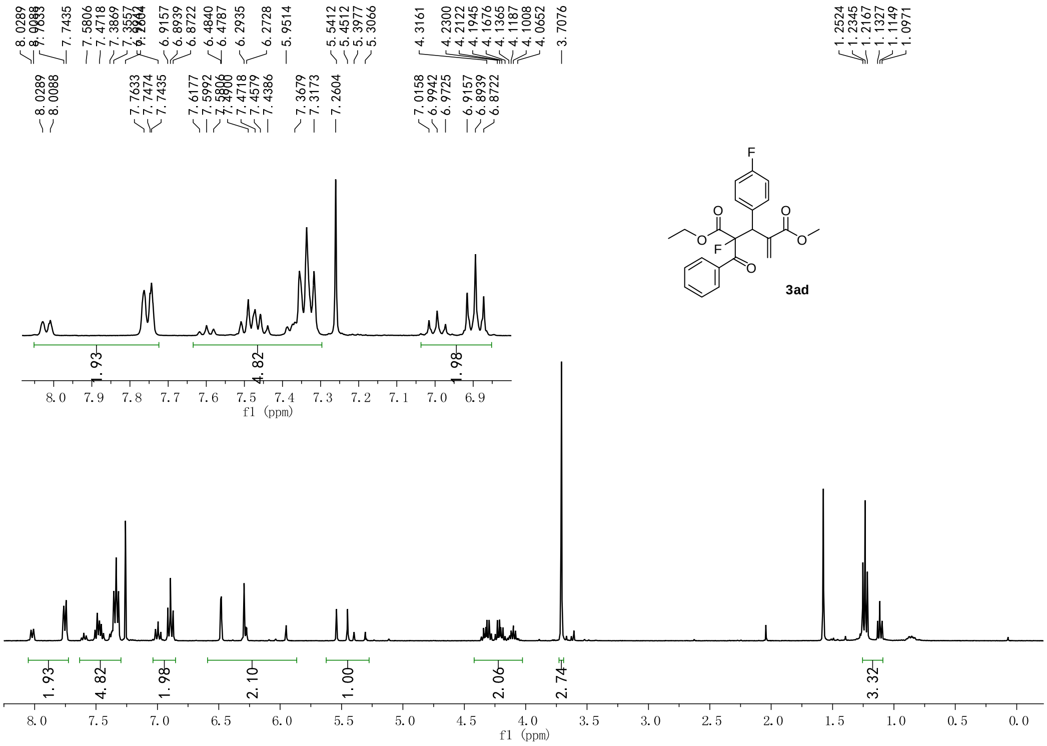

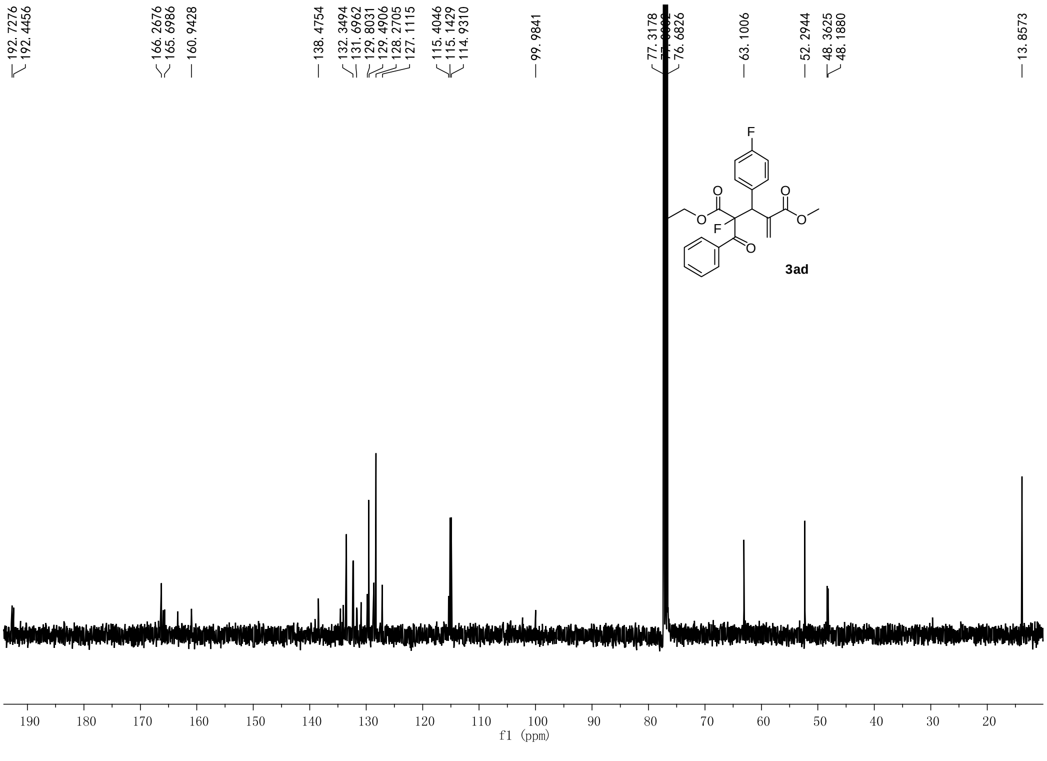

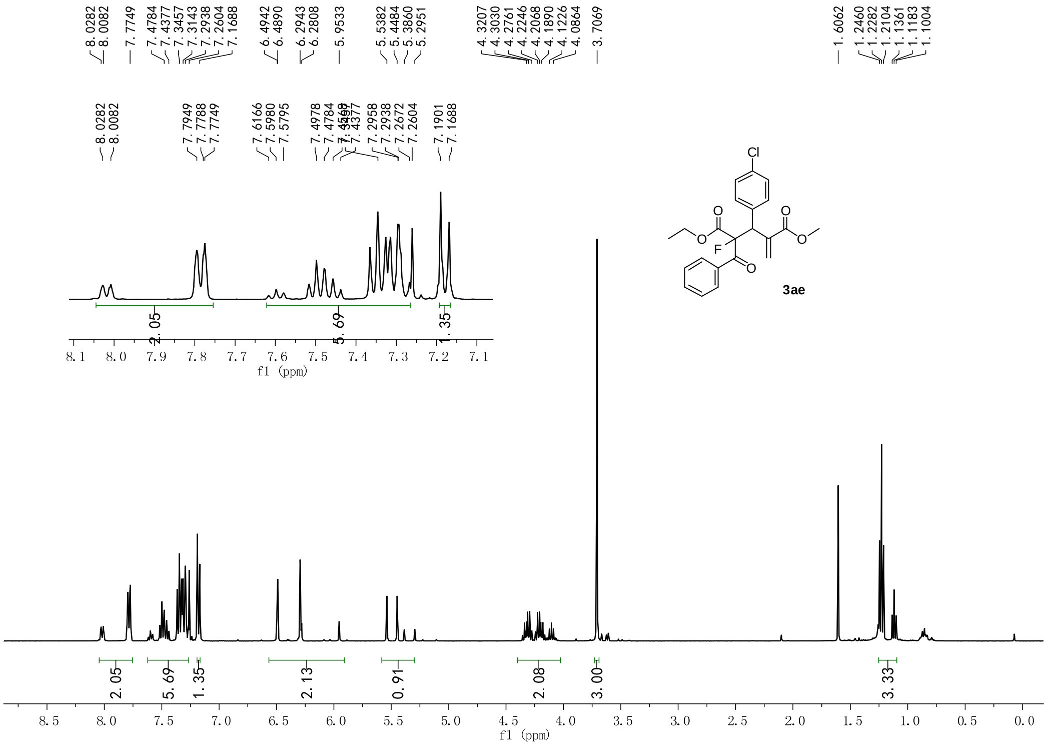
8.0282
8.0082
7.7749
7.4784
7.4377
7.3457
7.3143
7.2938
7.2604
7.1688
6.4942
6.4890
6.2943
6.2808
5.9533
5.5382
5.4484
5.3860
5.2951
4.3207
4.3030
4.2761
4.2246
4.2068
4.1890
4.1226
4.0864
3.7069
1.6062
1.2460
1.2282
1.2104
1.1361
1.1183
1.1004
8.0282
8.0082
7.7949
7.7788
7.7749
7.6166
7.5980
7.5795
7.4978
7.4784
7.4377
7.2958
7.2938
7.2672
7.2604
7.1901
7.1688
2.05
5.69
1.35
2.13
0.91
2.08
3.00
3.33
f1 (ppm)
3ae

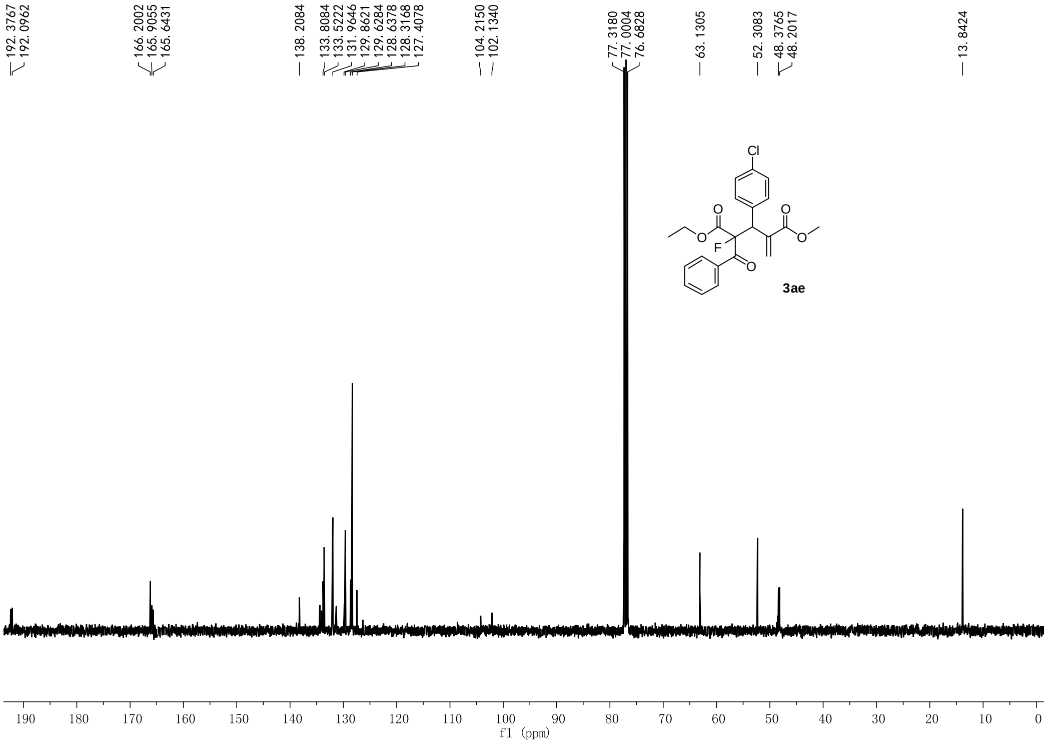
192.3767
192.0962
166.2002
165.9055
165.6431
138.2084
133.8084
133.5222
131.9646
129.8621
129.6284
128.6378
128.3168
127.4078
104.2150
102.1340
77.3180
77.0004
76.6828
63.1305
52.3083
48.3765
48.2017
13.8424
f1 (ppm)
3ae

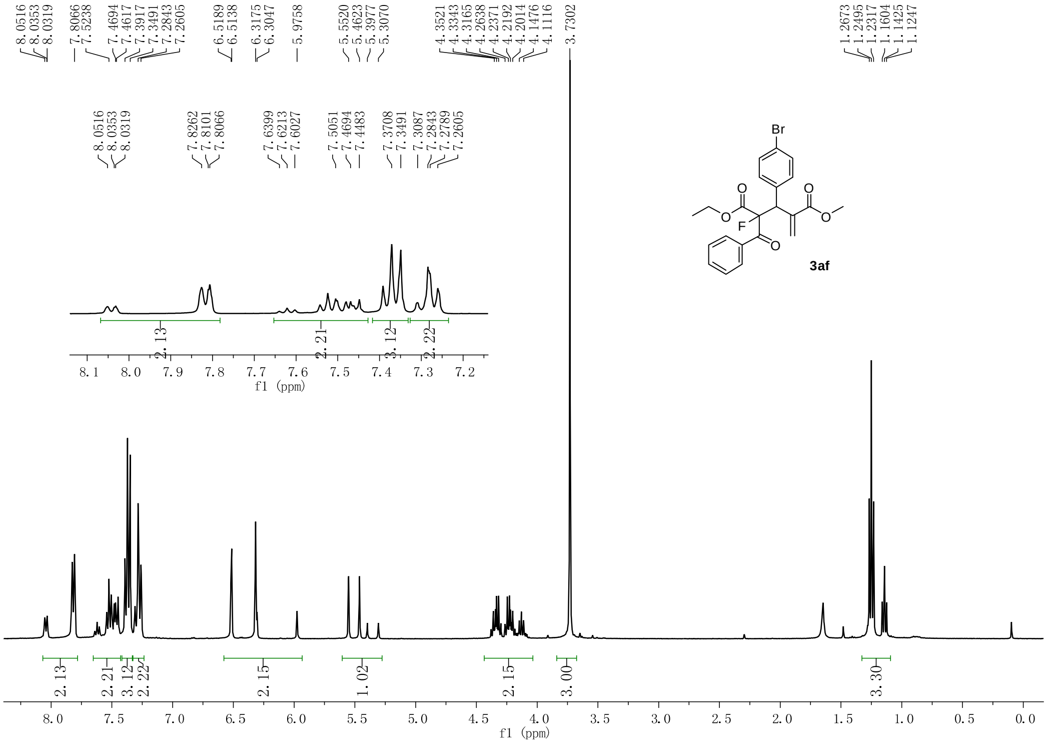

3af
Br
F
O
f1 (ppm)

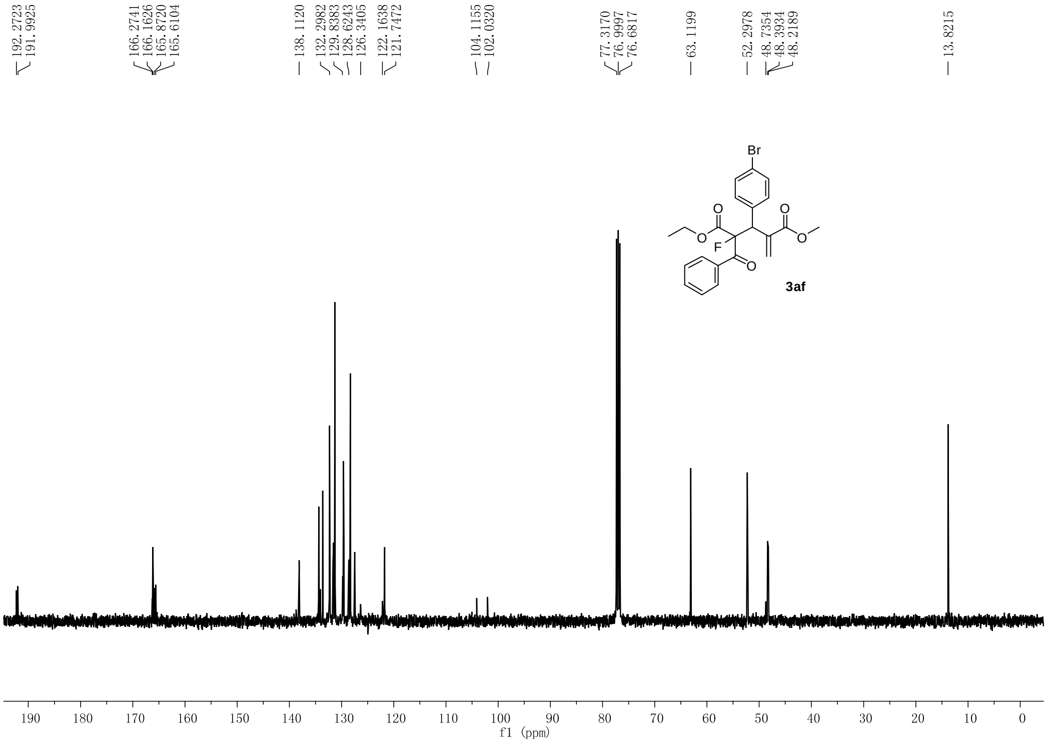

3af
Br
F
O
f1 (ppm)

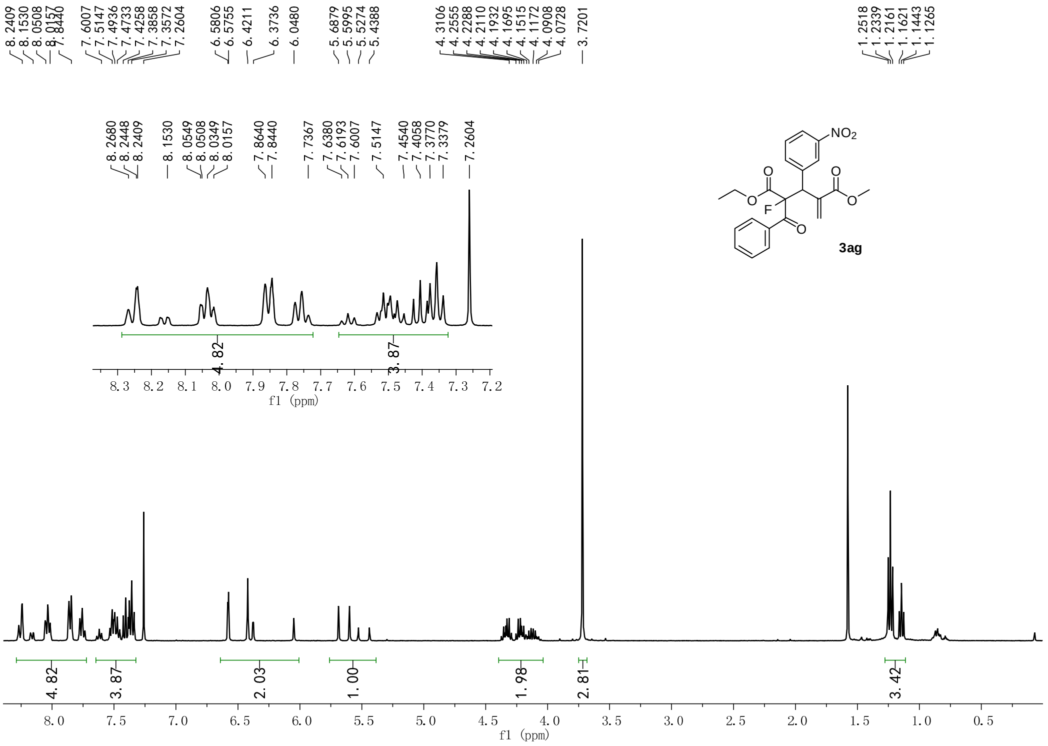
8.2409
8.1530
8.0508
8.0157
7.8440
7.6007
7.5147
7.4936
7.4733
7.4258
7.3858
7.3572
7.2604
6.5806
6.5755
6.4211
6.3736
6.0480
5.6879
5.5995
5.5274
5.4388
4.3106
4.2555
4.2288
4.2110
4.1932
4.1695
4.1515
4.1172
4.0908
4.0728
3.7201
1.2518
1.2339
1.2161
1.1621
1.1443
1.1265
8.2680
8.2448
8.2409
8.1530
8.0549
8.0508
8.0349
8.0157
7.8640
7.8440
7.7367
7.6380
7.6193
7.6007
7.5147
7.4540
7.4058
7.3770
7.3379
7.2604
4.82
3.87
f1 (ppm)
NO2
O
F
3ag
4.82
3.87
2.03
1.00
1.98
2.81
3.42
f1 (ppm)

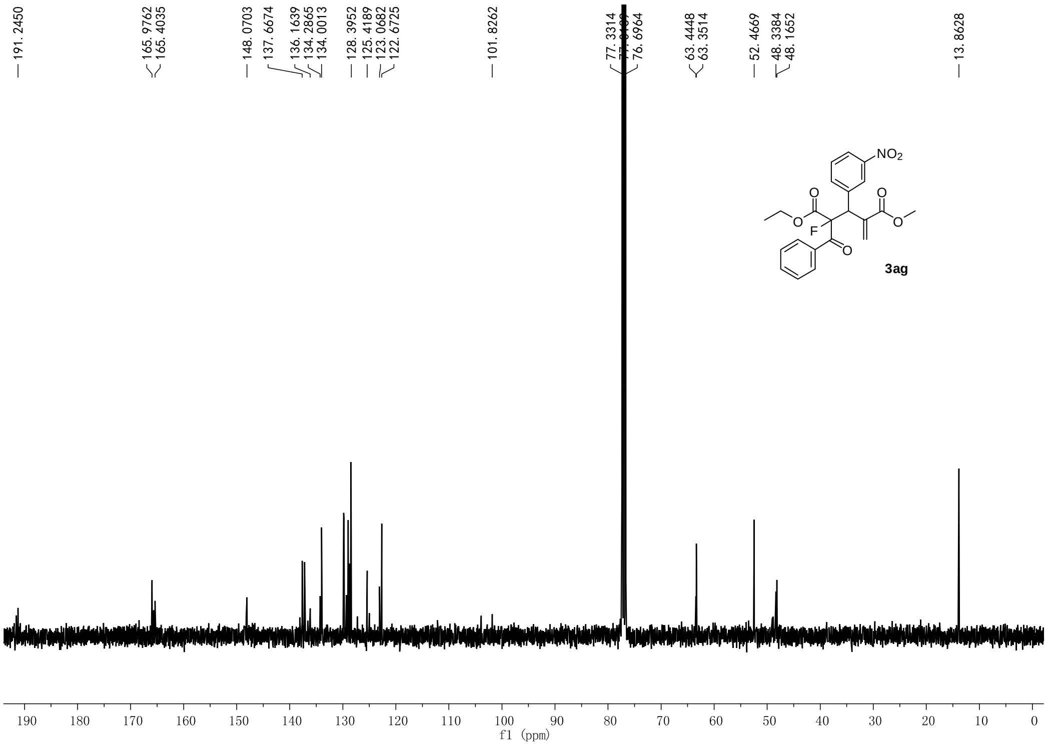
191.2450
165.9762
165.4035
148.0703
137.6674
136.1639
134.2865
134.0013
128.3952
125.4189
123.0682
122.6725
101.8262
77.3314
76.6964
63.4448
63.3514
52.4669
48.3384
48.1652
13.8628
NO2
O
F
3ag
f1 (ppm)

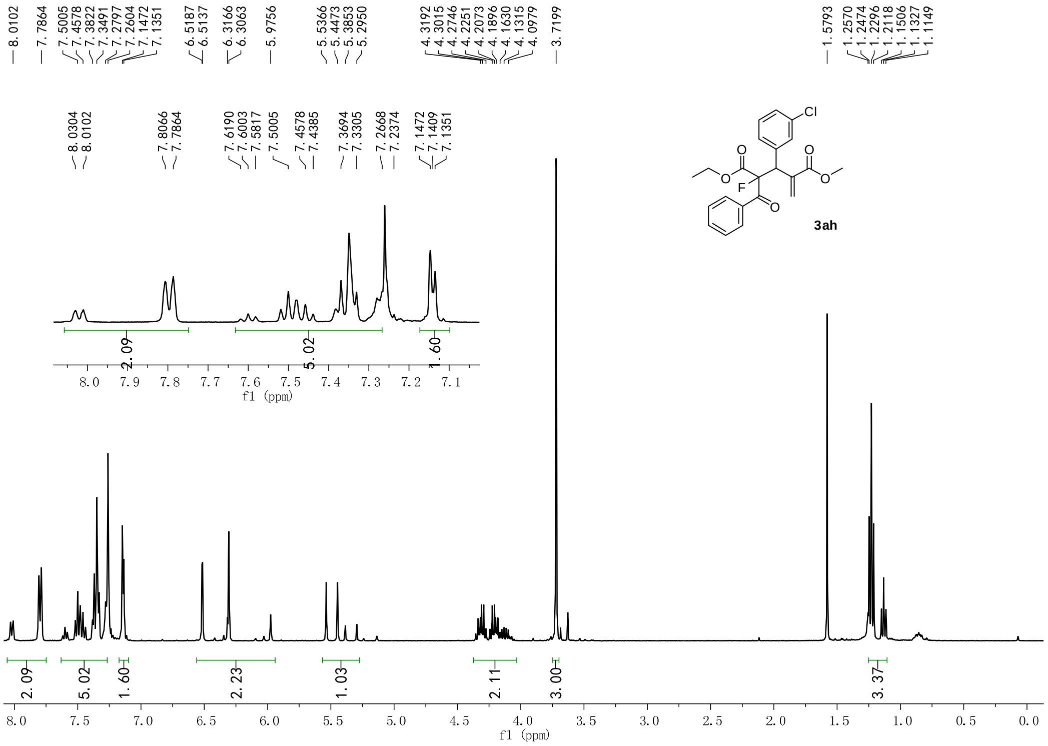
3ah
2.09
5.02
1.60
2.23
1.03
2.11
3.00
3.37
f1 (ppm)

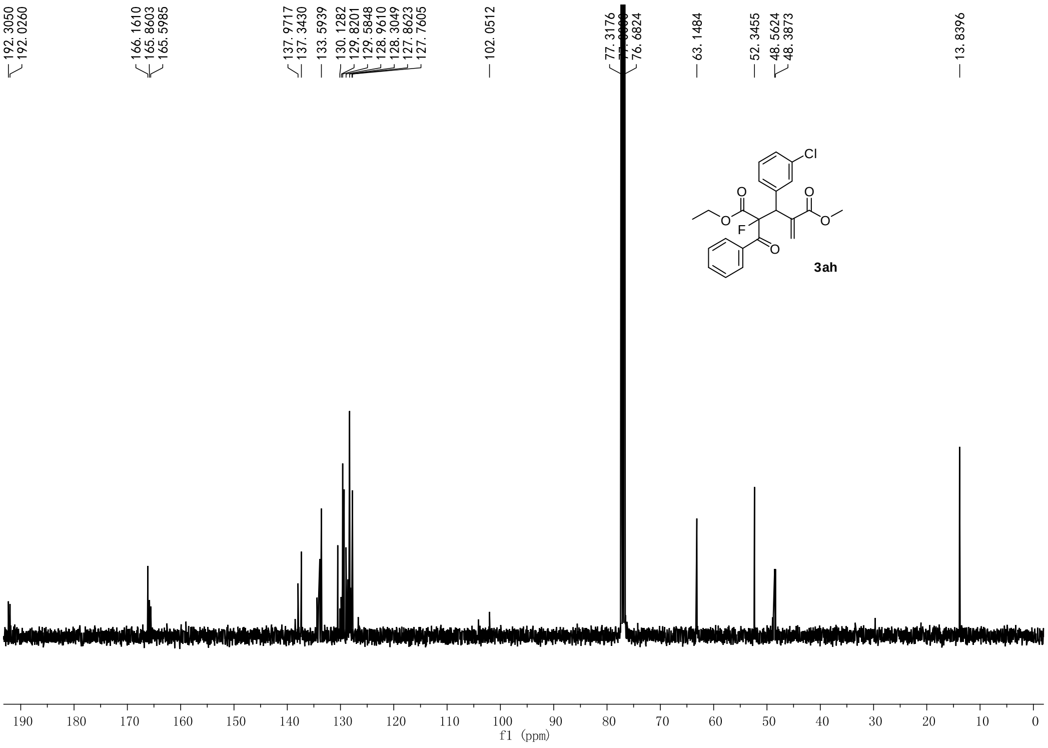
192.3050
192.0260
166.1610
165.8603
165.5985
137.9717
137.3430
133.5939
130.1282
129.8201
129.5848
128.9610
128.3049
127.8623
127.7605
102.0512
77.3176
76.6824
63.1484
52.3455
48.5624
48.3873
13.8396
3ah
f1 (ppm)

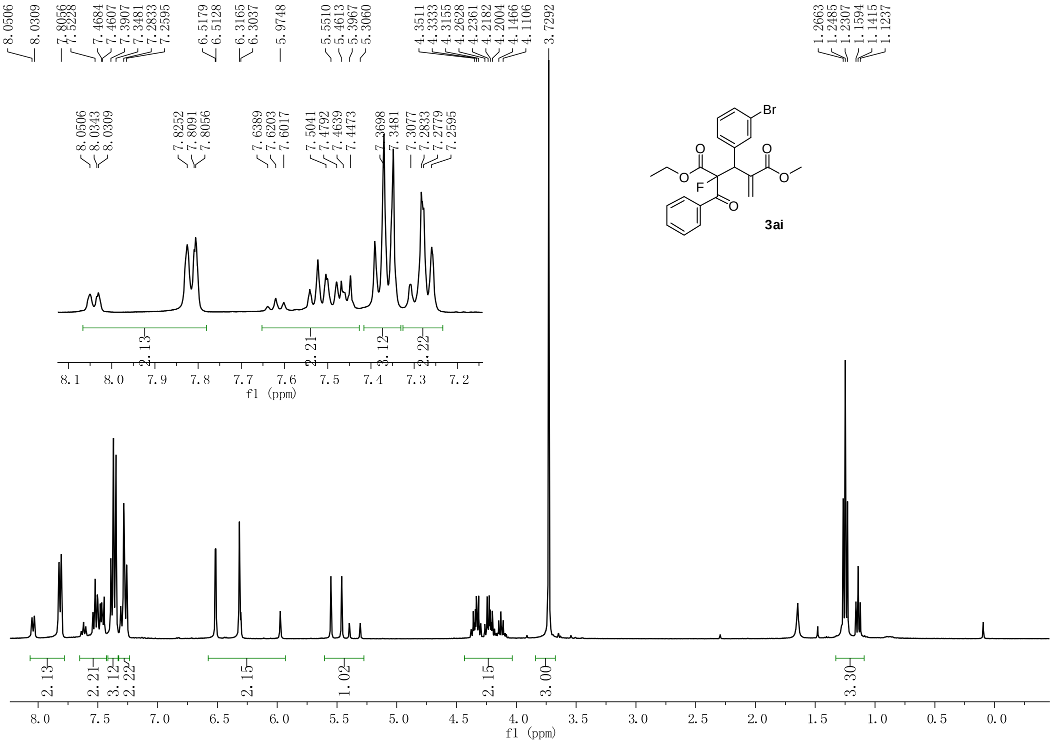
Br
F
O
3ai
f1 (ppm)

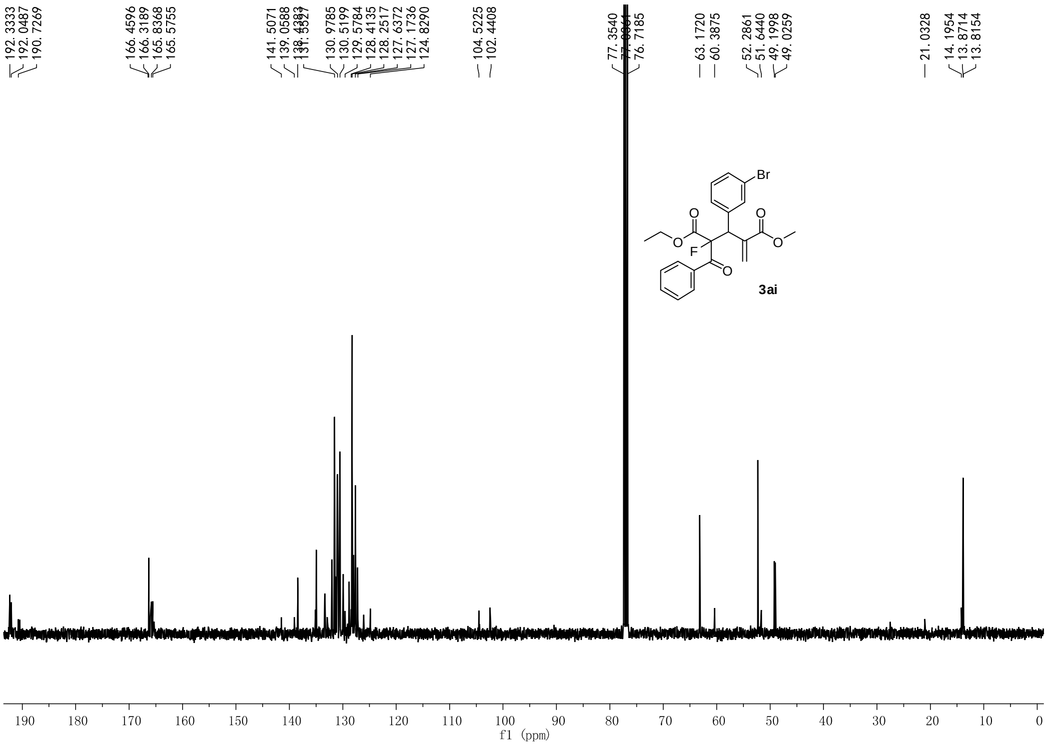
Br
F
O
3ai
f1 (ppm)

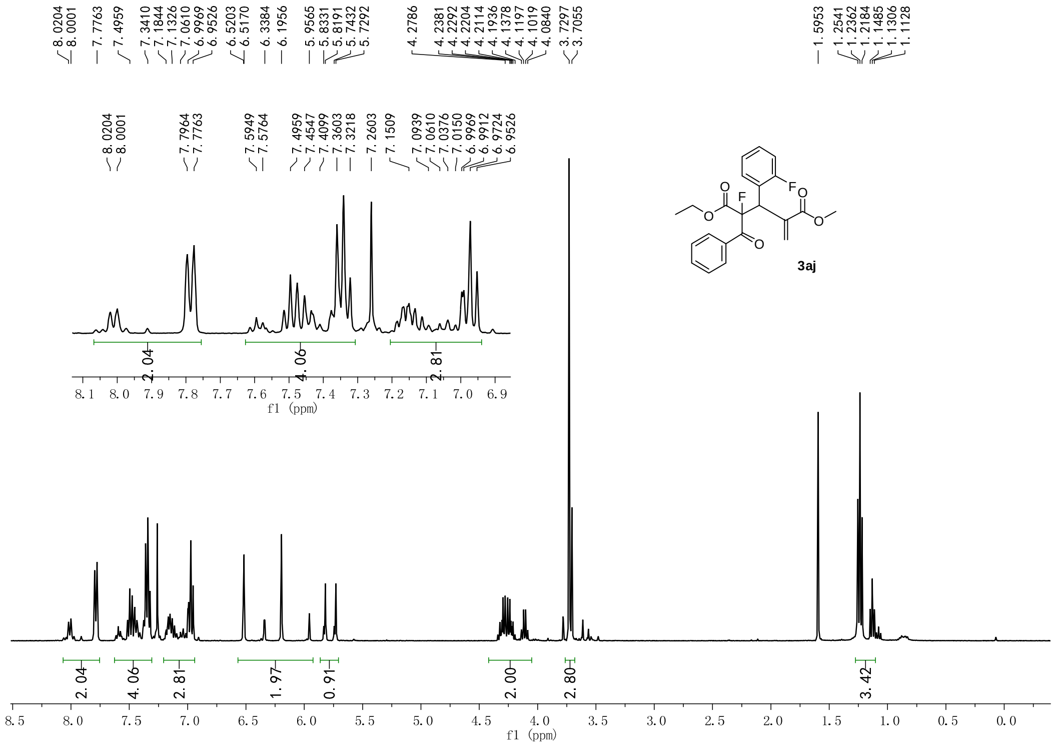
3aj
f1 (ppm)

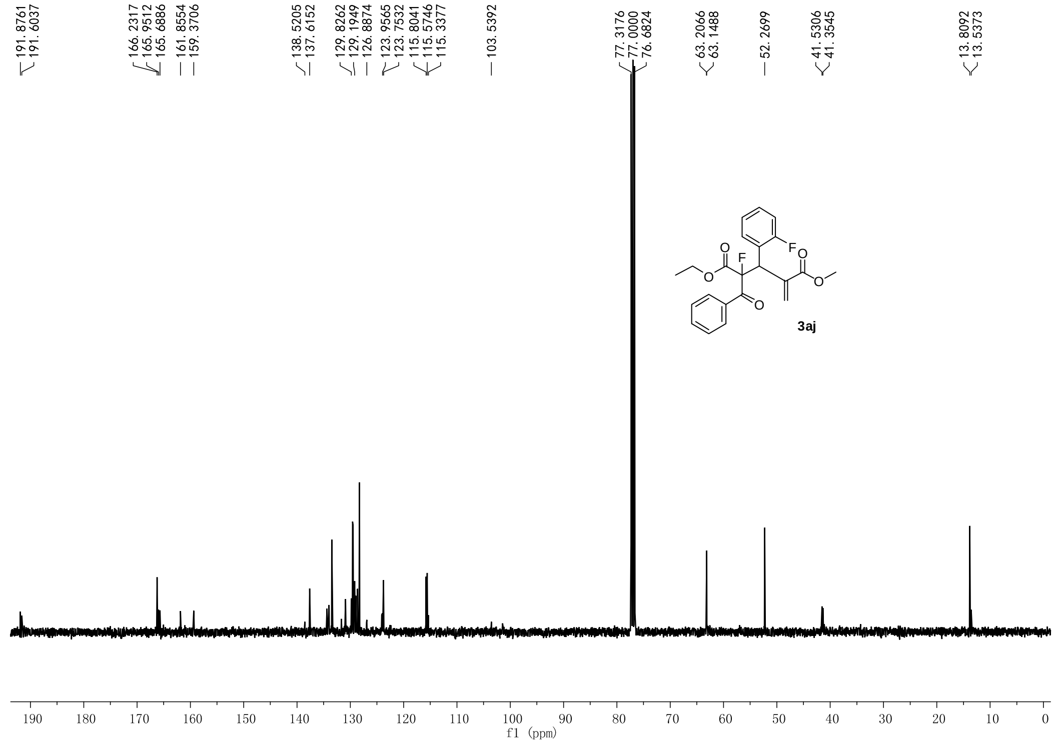
3aj
f1 (ppm)

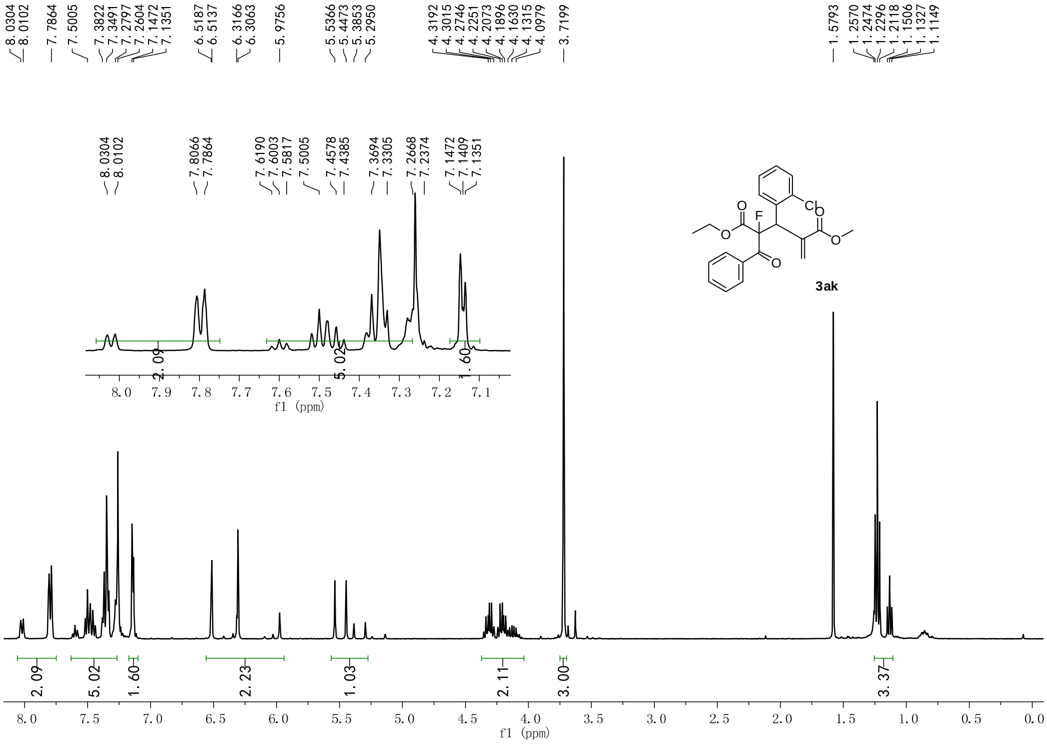

3ak

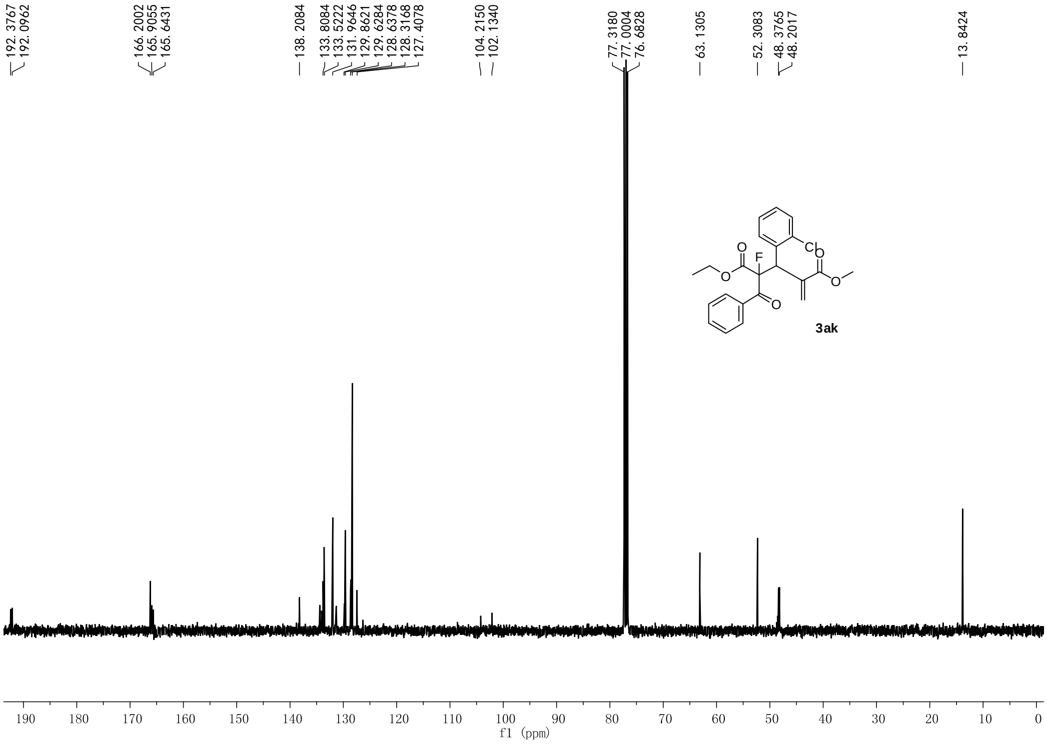

3ak

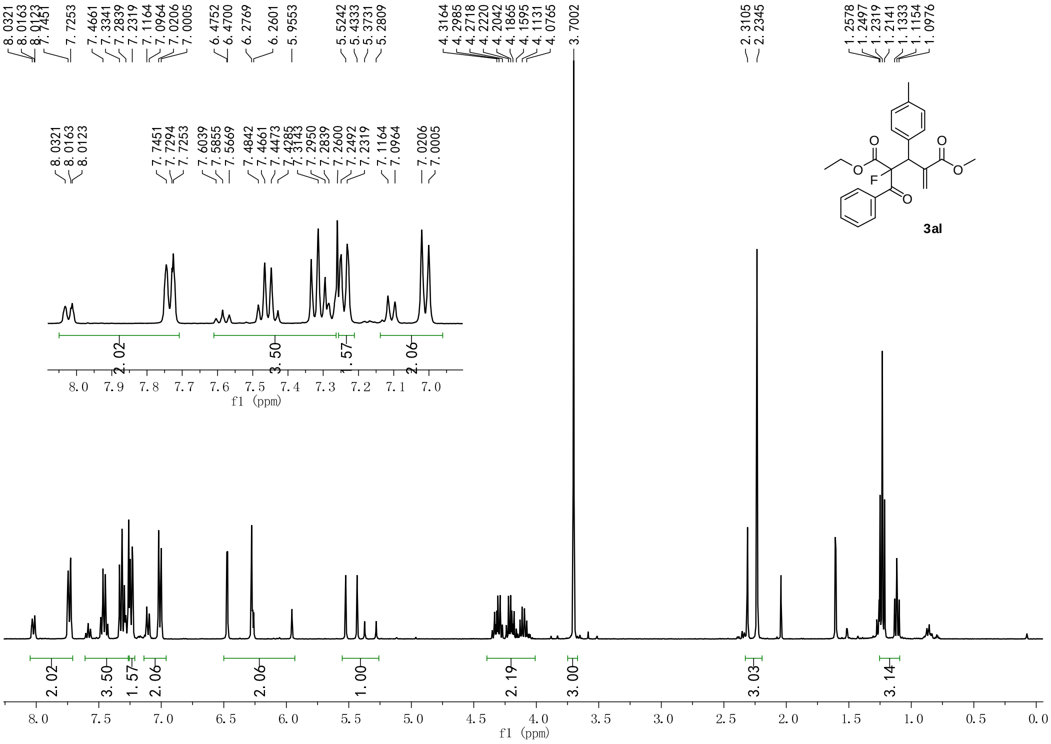
8.0321
8.0163
8.0123
7.7451
7.7253
7.4661
7.3341
7.2839
7.2319
7.1164
7.0964
7.0206
7.0005
6.4752
6.4700
6.2769
6.2601
5.9553
5.5242
5.4333
5.3731
5.2809
4.3164
4.2985
4.2718
4.2220
4.2042
4.1865
4.1595
4.1131
4.0765
3.7002
2.3105
2.2345
1.2578
1.2497
1.2319
1.2141
1.1333
1.1154
1.0976
8.0321
8.0163
8.0123
7.7451
7.7294
7.7253
7.6039
7.5855
7.5669
7.4842
7.4661
7.4473
7.4285
7.3143
7.2950
7.2839
7.2600
7.2492
7.2319
7.1164
7.0964
7.0206
7.0005
2.02
3.50
1.57
2.06
f1 (ppm)
F
O
3al
2.02
3.50
1.57
2.06
2.06
1.00
2.19
3.00
3.03
3.14
f1 (ppm)

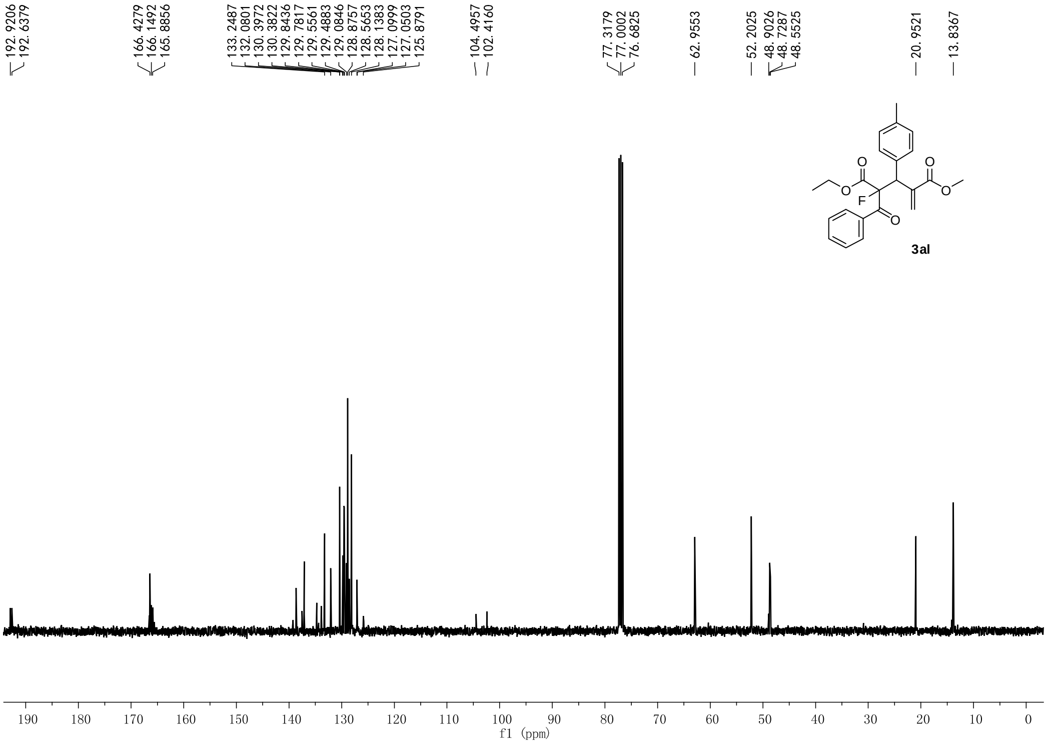
192.9206
192.6379
166.4279
166.1492
165.8856
133.2487
132.0801
130.3972
130.3822
129.8436
129.7817
129.5561
129.4883
129.0846
128.8757
128.5653
128.1383
127.0999
127.0503
125.8791
104.4957
102.4160
77.3179
77.0002
76.6825
62.9553
52.2025
48.9026
48.7287
48.5525
20.9521
13.8367
F
O
3al
f1 (ppm)

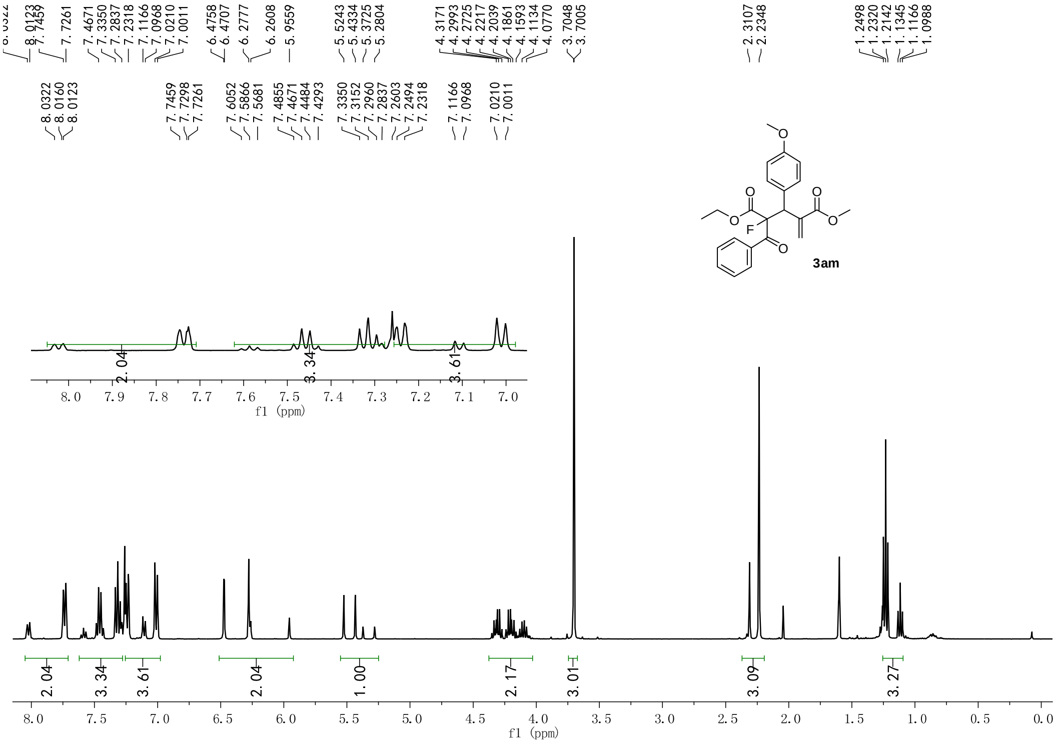

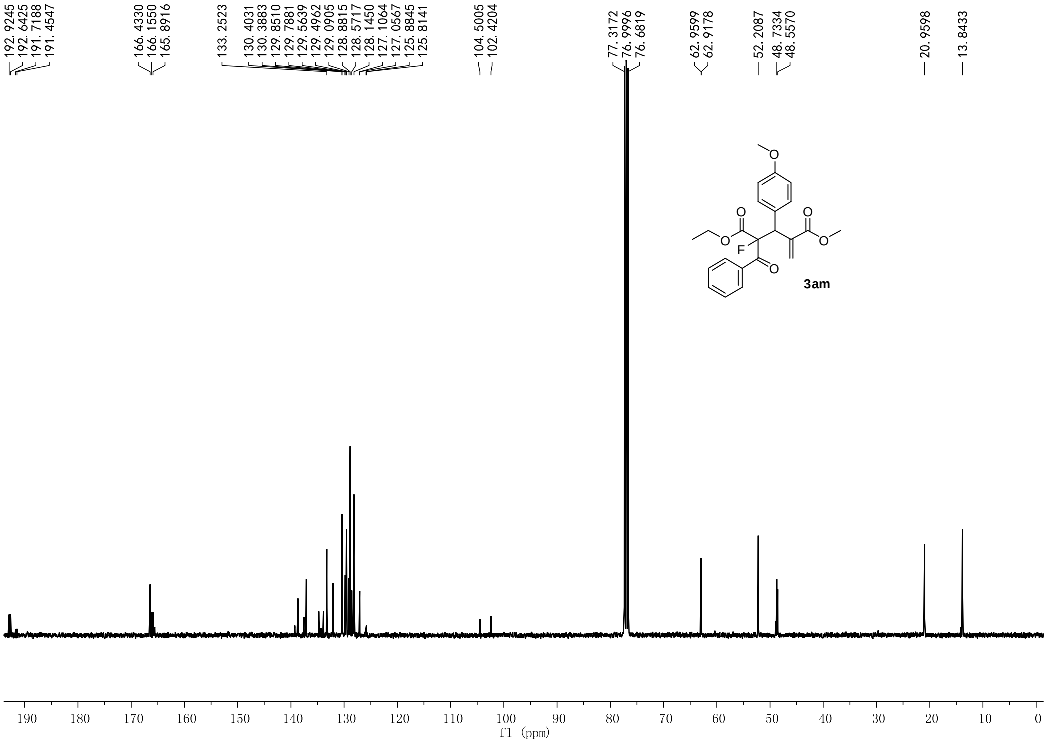

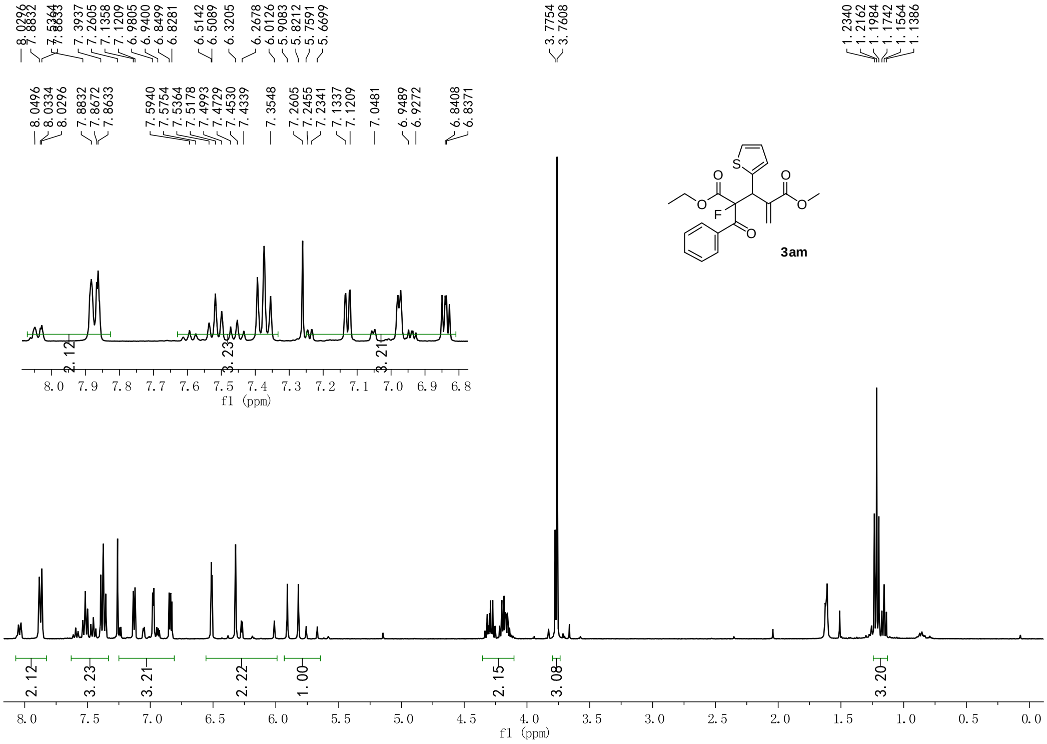

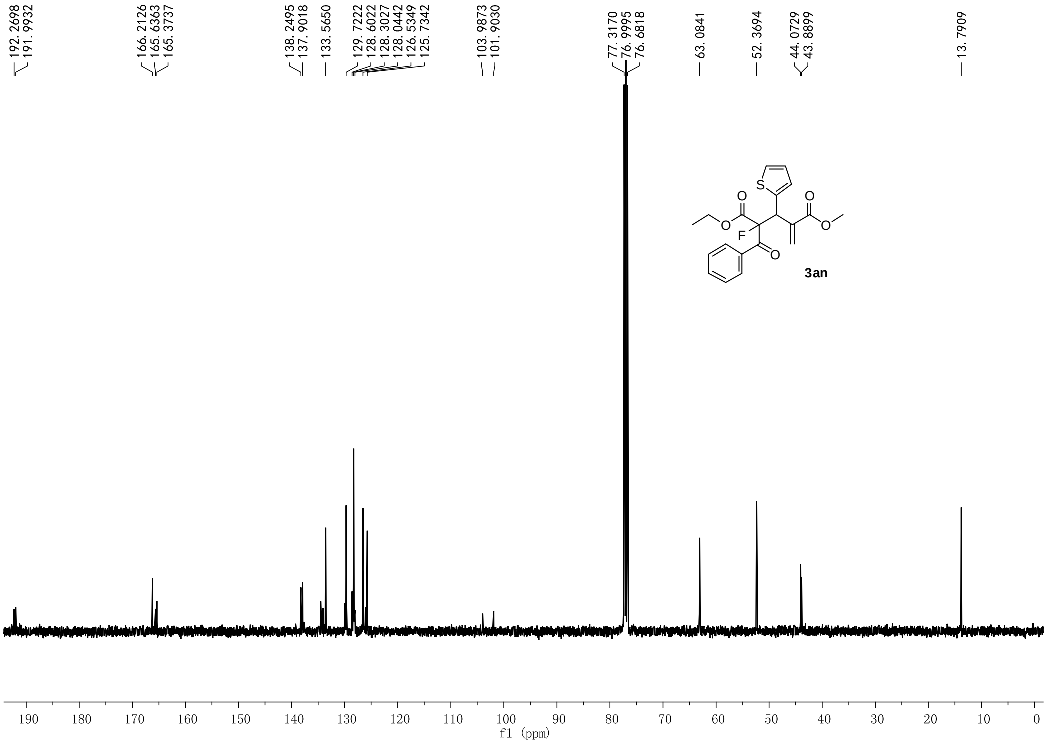

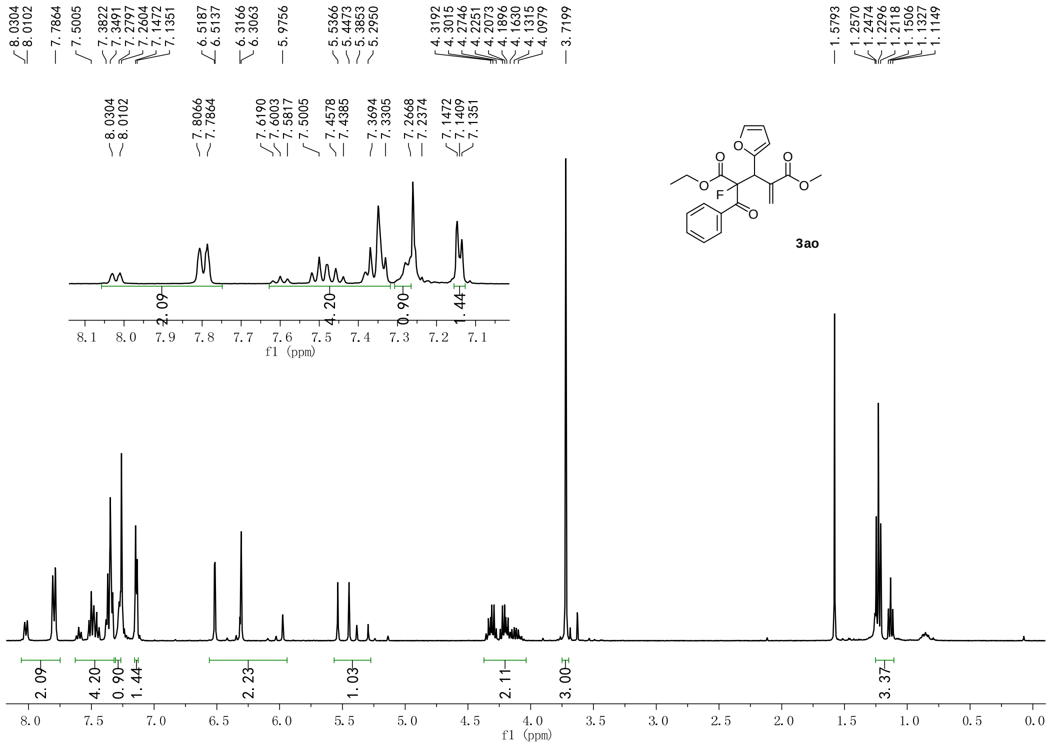
8.0304
8.0102
7.7864
7.5005
7.3822
7.3491
7.2797
7.2604
7.1472
7.1351
6.5187
6.5137
6.3166
6.3063
5.9756
5.5366
5.4473
5.3853
5.2950
4.3192
4.3015
4.2746
4.2251
4.2073
4.1896
4.1630
4.1315
4.0979
3.7199
1.5793
1.2570
1.2474
1.2296
1.2118
1.1506
1.1327
1.1149
3ao
f1 (ppm)

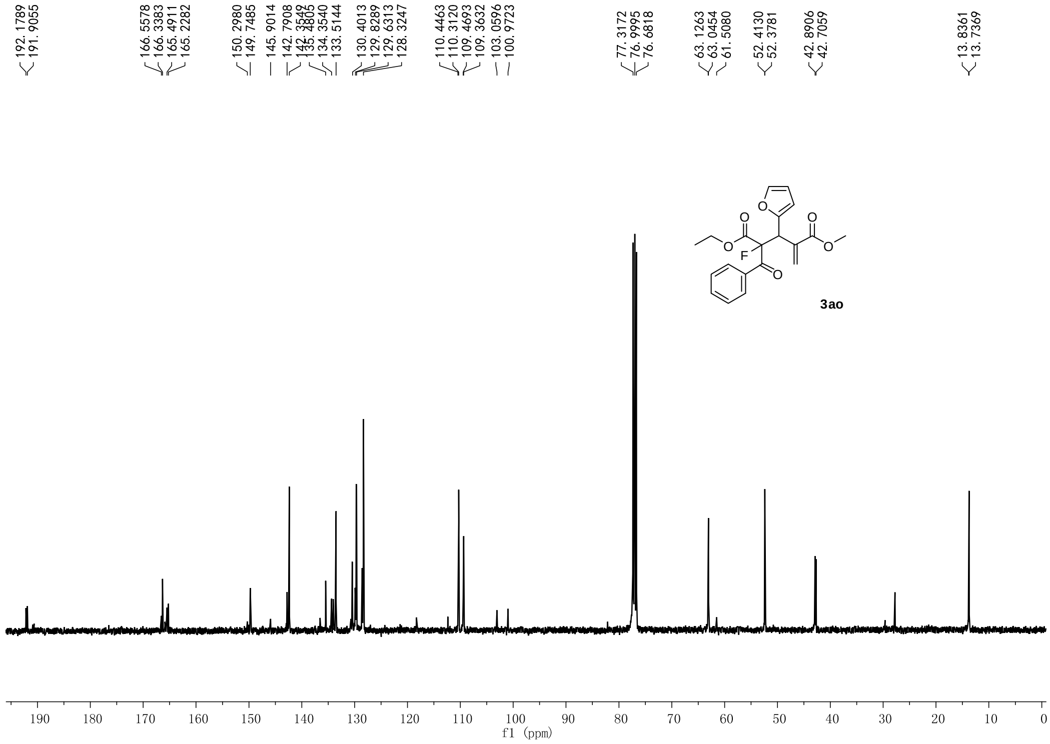
192.1789
191.9055
166.5578
166.3383
165.4911
165.2282
150.2980
149.7485
145.9014
142.7908
130.4013
129.8289
129.6313
128.3247
110.4463
110.3120
109.4693
109.3632
103.0596
100.9723
77.3172
76.9995
76.6818
63.1263
63.0454
61.5080
52.4130
52.3781
42.8906
42.7059
13.8361
13.7369
3ao
f1 (ppm)
